# Histone lysine methylation patterns in prostate cancer microenvironment infiltration: Integrated bioinformatic analysis and histological validation

**DOI:** 10.3389/fonc.2022.981226

**Published:** 2022-09-28

**Authors:** Yongjun Quan, Xiaodong Zhang, Mingdong Wang, Hao Ping

**Affiliations:** ^1^ Department of Urology, Beijing Tongren Hospital, Capital Medical University, Beijing, China; ^2^ Department of Urology, Beijing Chaoyang Hospital, Capital Medical University, Beijing, China; ^3^ Beijing Advanced Innovation Center for Big Data-Based Precision Medicine, Beihang University & Capital Medical University, Beijing Tongren Hospital, Beijing, China

**Keywords:** prostate cancer, histone lysine methylations, The Cancer Genome Atlas (TCGA), recurrence-free survival, HLMcluster, geneCluster, HLMscore

## Abstract

**Background:**

Epigenetic reprogramming through dysregulated histone lysine methylation (HLM) plays a crucial role in prostate cancer (PCa) progression. This study aimed to comprehensively evaluate HLM modification patterns in PCa microenvironment infiltration.

**Materials and methods:**

Ninety-one HLM regulators in The Cancer Genome Atlas (TCGA) dataset were analyzed using bioinformatics. Differentially expressed genes (DEGs) and survival analyses were performed using TCGA-PRAD clinicopathologic and follow-up information. Consensus clustering analysis divided patients into subgroups. Gene ontology (GO) function and Kyoto Encyclopedia of Genes and Genomes (KEGG) pathway enrichment analyses were performed on the DEGs. Tumor mutation burden (TMB) and tumor microenvironment (TME) cell infiltration were evaluated in different HLM clusters. Quantitative real-time PCR (qPCR) analysis assessed HLM regulators in clinical PCa tissues.

**Results:**

The tumor vs. normal (TN), Gleason score (GS) > 7 vs. GS < 7, pathological T stage (pT) = 2 vs. pT = 3, and TP53 mutation vs. wild-type comparisons using TCGA-PRAD dataset revealed 3 intersecting HLM regulators (EZH2, NSD2, and KMT5C) that were consistently upregulated in advanced PCa (GS > 7, pT3, HR > 1, and TP53 mutation) (P < 0.05) and verified in clinical PCa tissues. Consensus clustering analysis revealed three distinct HLM modification patterns (HLMclusters). However, no significant differences in recurrence-free survival (RFS) rates were found among the groups (P > 0.05). We screened 189 HLM phenotype-related genes that overlapped in the pairwise comparisons of HLMclusters and P < 0.01 in the Cox regression analysis. Three distinct subgroups (geneClusters) were revealed based on the 189 genes, in which cluster A involved the most advanced PCa (PSA > 10, T3-4, GS8-10, and biochemical recurrence) and the poorest RFS. The HLM score (HLMscore) was calculated by principal component analysis (PCA) of HLM phenotype-related genes that have positive predictive value for RFS (P < 0.001) and immune therapy responses (in the CTLA4-positive and -negative responses accompanied by a PD1-negative response).

**Conclusion:**

We comprehensively evaluated HLM regulators in the PCa microenvironment using TCGA-PRAD, revealing a nonnegligible role of HLM patterns in PCa complexity and heterogeneity. Elucidating the effects of HLM regulators in PCa may enhance prognostics, aggressiveness assessments, and immunotherapy strategies.

## Introduction

Prostate cancer (PCa) is a common malignancy among men over the age of 50 (1). In 2022, there were 268,490 (27%; the highest incidence among men with all cancers) estimated new PCa cases and 34,500 (11%; the second most common cause of all cancer-related deaths among men) estimated PCa-related in the United States (1). PCa progresses to castration-resistant PCa (CRPC) after androgen-deprivation therapy (ADT); CRPC is more aggressive and can be resistant to subsequent chemotherapy, ultimately leading to cancer-related death. Therefore, identifying the molecular mechanisms associated with PCa progression is crucial for diagnosis, risk prediction, and treatment.

Nucleosome core particles can posttranslationally modify histones to maintain genomic integrity (2–4). Well-known histone modifications include methylation, acetylation, phosphorylation, and ubiquitylation, which alter the binding force of DNA to histones or recruit specific histone-binding proteins to epigenetically regulate genomic activity (4–6). Among these modifications, histone methylation is associated with heterochromatin formation and the regulation of target gene promoter activity (7, 8). Histone modifications occur on mainly the positively charged lysine (H3(K4, 9, 27, 36, and 79) and H4K20) and arginine (H3 (R2, 8, 17, and 26) and H4R3) residues in the N-terminal tails of wrapped DNA (9, 10). The histone lysine methylation (HLM) sites are highly conserved and precisely balanced by histone methyltransferases (HMT, “writers”), demethylases (HDM, “erasers), and methyl-lysine-recognizing proteins (MLRP, “readers”) (11–13).

Epigenetic reprogramming through inhibition of the enhancer of zeste homolog 2 (EZH2) has been found to enhance the effectiveness of enzalutamide (ENZ) treatment in CRPC patients (14, 15). EZH2 is a histone H3 lysine 27 (H3K27Me3) methyltransferase that silences the transcription of target genes. EZH2 has been found to be highly expressed in CRPC and neuroendocrine PCa (NEPC), and inhibition of EZH2 induces androgen receptor (AR) signaling reactivation in CRPC and further sensitivity to ADT (16–19). Studies of > 100 patients have indicated that EZH2 is a potential valuable, powerful prognostic parameter for PCa progression before or after treatment (20, 21). Authoritative studies have also elucidated that other HLM regulators, such as NSD2, SMYD3, LSD1, and DOT1L, play crucial roles in PCa progression by coordinating with transcription factors (such as AR and FOXA1) and performing the functions of histone methylation (22–27). Our previous study revealed that MLL5α (a smaller isoform of KMT2E) can prevent PCa progression by promoting AR/NDRG1 signaling *via* histone methylation (28). This finding implies a critical role for HLM regulators in PCa progression.

Nevertheless, few studies have focused on HLM modification patterns in different PCa risk stages and prognoses. Here, we performed a comprehensive transcriptome and genomic study of acknowledged HLM regulators (51 writers, 21 erasers, and 19 readers) in PCa by conducting bioinformatic analysis and using clinical PCa samples.

## Materials and methods

### Data acquisition

The data category of The Cancer Genome Atlas (TCGA) in transcriptome profiling, simple nucleotide variation, copy number variation (CNV), and clinical phenotype were downloaded from the Genomic Data Commons (GDC) Data Portal (https://portal.gdc.cancer.gov/) and University of California Santa Cruz Xena (UCSC) browser (https://xena.ucsc.edu/) as described in our previous study (29).

### Differentially expressed gene (DEG) analysis

Transcripts per kilobase of exome per million (TPM) mapped reads were transformed from the transcriptome profiling data of HTSeq-FPKM. DEGs in variant phenotypes of PCa and normal prostate tissues were analyzed through the Wilcoxon or Kruskal−Wallis tests using the R packages “limma”, “reshape2”, and “ggpurb”.

### Enrichment analysis

The DEG-related functional and signaling pathways were evaluated through gene ontology (GO) function and Kyoto Encyclopedia of Genes and Genomes (KEGG) pathway enrichment analyses utilizing the R packages “clusterProfiler” and “enrichplot”.

### Survival and correlation analyses

Recurrence-free survival (RFS) analyses were performed based on the clinical phenotype data of “days_to_first_biochemical_recurrence” and “days_to_last_follow_up.diagnoses”, which we previously described (29). The survival curves were generated *via* the Kaplan–Meier method, and statistical significance was evaluated through log-rank tests. Univariate Cox regression analysis was conducted based on the DEGs of HLM clusters (HLMclusters), and P < 0.01 was used for subsequent gene consensus clustering analysis. The survival analyses were implemented utilizing the R packages “survival” and “survminer”. Pearson or Spearman correlation analyses were performed, and a prognostic network map was drawn utilizing the R packages “igraph”, “psych”, “reshape2”, “RColorBrewer”, “corrplot”, and “ggpubr”.

### Consensus clustering analysis and principal component analysis (PCA)

To evaluate the characteristics of classifying patterns of HLM modification phenotypes in the clinicopathological features and prognosis of PCa patients, TCGA-PRAD patients were divided into subgroups by conducting consensus clustering analysis. This method identified distinct HLM modification patterns based on the expression of HLM regulators or HLM phenotype-related genes. Consensus clustering analysis was applied using the R package “ConsensusClusterPlus”. The clustering results were graphically displayed as a heatmap of the consensus matrices, consensus cumulative distribution function (CDF) plots, and delta area plots. The number of clusters and their stability were determined as previously described (29–31), including the criteria of relatively high consistency within the cluster, low variation coefficient, and no appreciable increase in the area under the CDF curve. PCA was conducted to confirm the fitness and correctness of the HLM modification patterns using the pcromp function of R software.

### HLM signature generation

A set of scoring system (HLMscore) was constructed in PCa to quantify HLM modification patterns as previously described (31). The intersected HLM phenotype-related genes in the pairwise comparisons of three HLMclusters were extracted, and the significant prognostic genes were further screened *via* Cox regression models (P < 0.01). Then, PCA was conducted using screened genes to construct an HLM-relevant gene signature. Both principal component 1 (PC1) and component 2 (PC2) were regarded as signature scores. The HLMscore was calculated as follows:


$$HLMscore=\sum (PC1_{i}+PC2_{i})$$

where i = the expression of HLM phenotype-related genes.

### Gene set variation analysis (GSVA)

GSVA was performed to assess the potential dysfunctional pathways in different clusters. GSVA comprehensively scored the DEGs and transformed them into KEGG pathways (32). The “KEGG gene sets as Gene Symbols” were downloaded from the website of Gene Set Enrichment Analysis (GSEA) (http://www.gsea-msigdb.org/gsea/), and the GSVA algorithm was implemented through the R packages “GSEABase” and “GSVA” to score each gene set.

### Tumor mutational burden (TMB) estimation

TMB contributes to immune recognition of cancer and was calculated by the total number of somatic mutations per million bases (33). TCGA-PRAD patients were classified into low-TMB and high-TMB groups based on the TMB value of optimum threshold segmentation (lowest log-rank P value in the Kaplan‒Meier analysis) in the RFS analysis. The correlation between the HLM score (shown as HLMscore below) and TMB value was then analyzed.

### Tumor microenvironment (TME) cell infiltration

Sigle-sample gene set enrichment analysis (ssGSEA) was performed to quantify TME cell infiltration by calculating enrichment scores (32). The gene set related to immune cell type in each TME cell infiltration was obtained as previously reported (34, 35). Then, we quantitatively analyzed the immune cell type infiltration in different HLMclusters.

### Immunophenoscore (IPS) analysis

As reported previously, IPS determines immunogenicity and is calculated according to the expression levels of the genes in representative cell types (36). The IPSs of TCGA-PRAD were obtained from The Cancer Immunome Atlas (TCIA) website (https://tcia.at/home) and then statistically analyzed in different HLMscore groups.

### Clinical PCa samples

Forty-two PCa tissues (14 with Gleason score (GS) < 7, 14 with GS = 7, and 14 with GS > 7) and 14 adjacent normal tissues were collected from PCa patients in accordance with Ethics Committee guidelines. All patients underwent prostatectomy between 2016 and 2021 at Beijing Tongren Hospital and Beijing Chaoyang Hospital. **Table 1** summarizes the clinicopathological characteristics of the patients.

**Table 1:** Clinicopathological characteristics of clinical PCa patients.

Clinicopathological parameters	Total (n = 42) (%)
**Age**	
Median (IQR)	64.5 (59.0-70.5)
Range (min, max)	52-78
< 65	21 (50%)
≥ 65	21 (50%)
**Total PSA (t-PSA)**	(ng/ml)
Median (IQR)	13.34 (6.945-26.605)
Range (min, max)	2.17-92.21
< 4 ng/ml	2 (4.8%)
4-10 ng/ml	15 (35.7%)
10-20 ng/ml	10 (23.8%)
> 20 ng/ml	15 (35.7%)
**Gleason Score (GS)**	
< 7	14 (33.3%)
7	14 (33.3%)
> 7	14 (33.3%)
**Clinical T-stage**	
T2a	10 (23.8%)
T2b	15 (35.7%)
T2c	6 (14.3%)
T3a or T3b	11 (26.2%)
**Lymph node metastasis**	
N0	34 (81%)
N1	8 (19%)
**Distant metastasis**	
M0 or Mx	40 (95.2%)
M1	2 (4.8%)
**TNM stage**	
I-II	28 (66.7%)
III-IV	14 (33.3%)

### Total RNA extraction and quantitative real-time PCR (qPCR) analysis

Total RNA extraction and complementary DNA (cDNA) reverse transcription were performed using TRIzol™ reagent (Invitrogen, Carlsbad, CA, USA) and One-Step gDNA Removal and cDNA Synthesis SuperMix (TransGen Biotech, Beijing, China) according to the manufacturer’s instructions. qPCR was performed with Top Green qPCR SuperMix (TransGen Biotech) on an SDS 7500 FAST Real-Time PCR system (Applied Biosystems, Foster City, CA, USA). The endogenous reference genes 18S ribosomal RNA and GAPDH were used as controls. **Supplementary Table 1** (**Table S1**) shows the relevant primer sequences.

### Statistical analysis

R software (4.0.3), SPSS software version 23 (IBM, Armonk, New York, USA), Microsoft Excel 2019 software (Microsoft Corp., Redmond, WA, USA), and GraphPad Prism 7.0 (GraphPad Software, La Jolla, CA, USA) were used to conduct the statistical analyses. Continuous data were analyzed using Wilcoxon or Kruskal–Wallis tests. Pearson and Spearman correlation analyses were used to determine the association between two variables. Kaplan–Meier survival analyses with a log-rank test and univariate Cox regression models with a hazard ratio (HR) were utilized for the survival analyses.

## Results

### Workflow of this study

**Figure 1:**
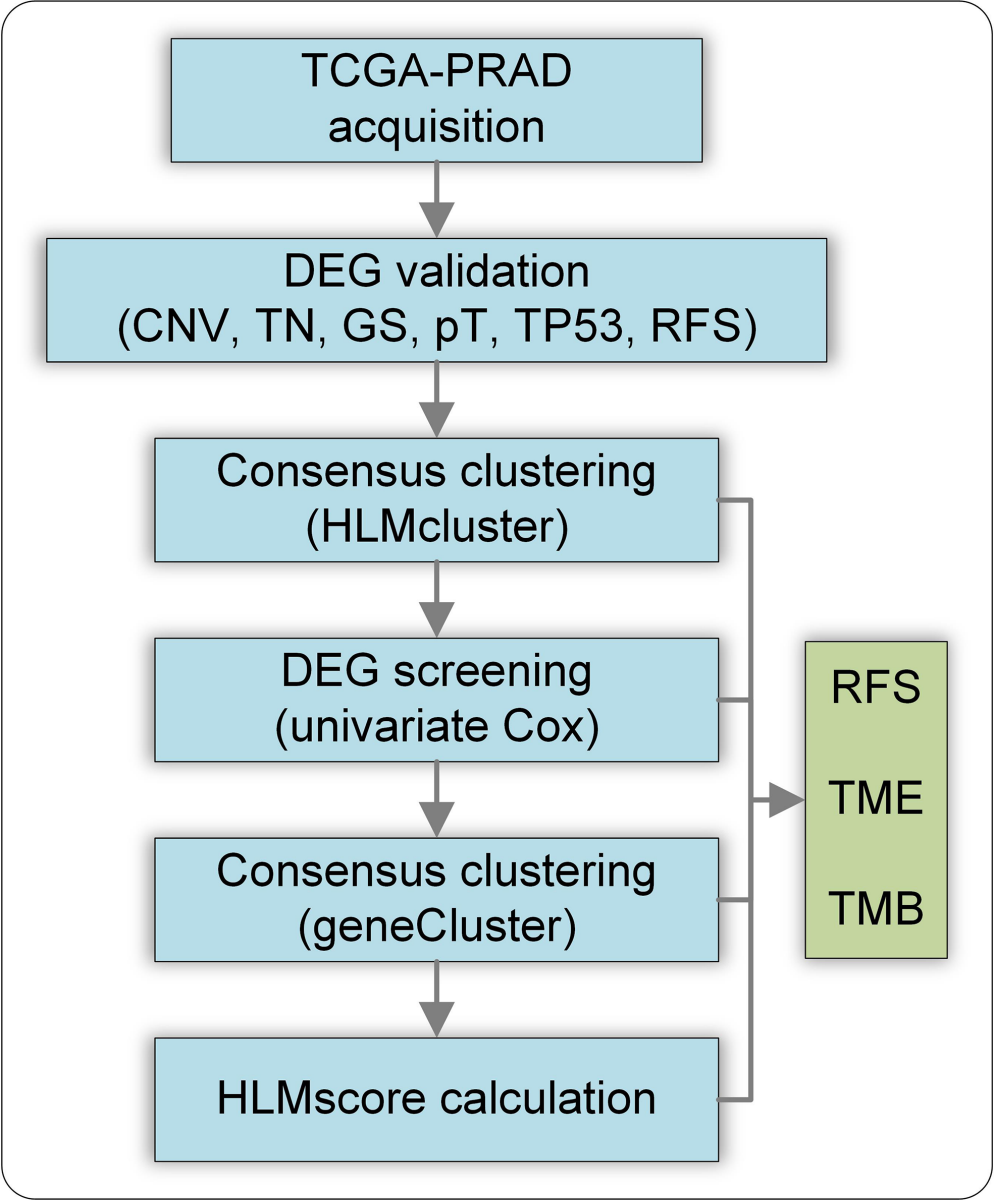
Flow chart of this study. CNV, CNV loss or gain; TN, tumor vs. normal; GS, GS >7 vs. GS< 7; pT, pT3 vs. pT2 (pT); TP53, TP53 mutation vs. wild type; RFS, RFS high vs. low expression of genes.

This study was conducted using CNV and TPM mapped reads in the TCGA-PRAD dataset. The flow chart of this study is shown in **Figure 1**. The DEGs of HLM regulators in TCGA-PRAD data stratified by CNV loss or gain (CNV), tumor vs. normal (TN), GS > 7 vs. GS < 7 (GS), pT3 vs. pT2 (pT), TP53 mutation vs. wild type (TP53), and RFS high vs. low expression were analyzed, and the intersecting genes were validated using clinical PCa tissues. Then, the TCGA-PRAD patients were divided into three groups (HLMcluster) based on 91 HLM regulators *via* consensus clustering analysis. The HLM phenotype-related genes overlapped in the pairwise comparisons of HLMclusters were screened *via* univariate Cox regression analysis. TCGA-PRAD patients were further divided into three groups (geneCluster) according to the expression of screened genes, and the HLMscore was calculated through PCA of the HLM phenotype-related genes. The clusters (HLMclusters and geneClusters), screened genes, and HLMscores were evaluated using RFS, TMB, and TME analyses.

### Characteristics of HLM regulators in PCa

**Figure 2:**
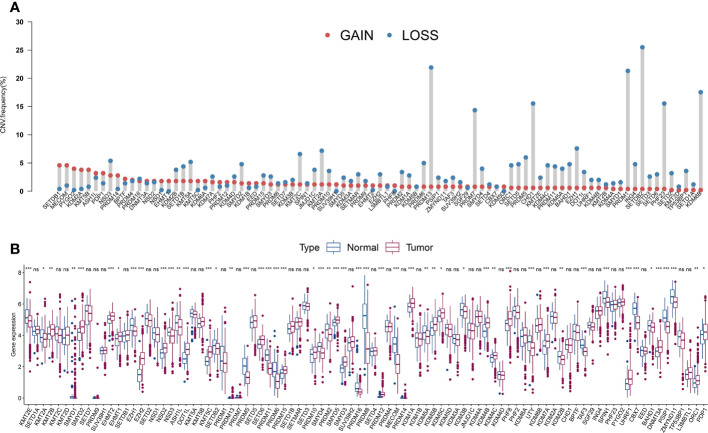
The CNV variation frequency and the expression levels of HLM regulators in PCa. **(A)** The CNV variation frequency of HLM regulators in TCGA-PRAD. Red dot, amplification frequency; Blue dot, deletion frequency. **(B)** The expression levels of the 91 HLM regulators in PCa and normal prostate tissues are shown as boxplot. The values of median ± interquartile ranges are shown in the graph. ns P > 0.05; *P < 0.05; **P < 0.01; ***P < 0.001.

The average estimated CNVs were previously considered a method to determine PCa malignancy (37). We analyzed the CNV alteration frequency and DEGs in HLM regulators in TCGA-PRAD and normal tissues. Regarding CNV events, approximately 7.87% (7 of 89) of HLM regulators had widespread CNV deletion levels (> 10%), among which SETDB2 (25.50%), PRDM13 (21.91%), and PRDM1 (21.31%) had the three highest degrees of copy number loss. All HLM regulators had a low proportion of CNV amplification (< 5%), with MECOM (4.58%) and SETDB1 (4.58%) having the highest CNV gain (**Figure 2A**). The chromosomal location and CNV alterations in HLM regulators are shown in **Figure S1A**. DEGs in HLM regulators in PCa and normal prostate tissues were statistically analyzed using the TPM dataset of TCGA-PRAD. Twenty-six HLM regulator genes with significantly higher expression levels (including EZH2, PRDM12, and KMT5C) and 25 with lower expression levels (including MECOM, PRDM11, CBX7, and so on) were found in PCa tissues compared with normal prostate tissues (P < 0.05) (**Figure 2B** and **Table S2**). Unlike the findings in a previous study of gastric cancer (31), no significant correlation between CNV alteration and DEG expression was found in TCGA-PRAD. In the mutation frequency analysis, we found that only 58 gene symbols of HLM regulators transformed from the “ensemble ID” were matched with the gene symbol in the TCGA-PRAD data category of “simple nucleotide variation” and data type of “Masked Somatic Mutation”. Among the 58 identified HLM regulators, mutations were found in 112 of 484 samples (23.14%), among which KMT2D (24 of 484), KMT2C (20 of 484), and KDM6A (11 of 484) were the most frequently mutated genes in TCGA-PRAD (**Figure S1B**). Nevertheless, most of the genes exhibited low mutation rates (≤ 1%), implying highly conserved and stable expression levels of HLM regulators in PCa.

### The expression levels of HLM regulators related to various clinicopathological characteristics and the prognosis of PCa patients

**Figure 3:**
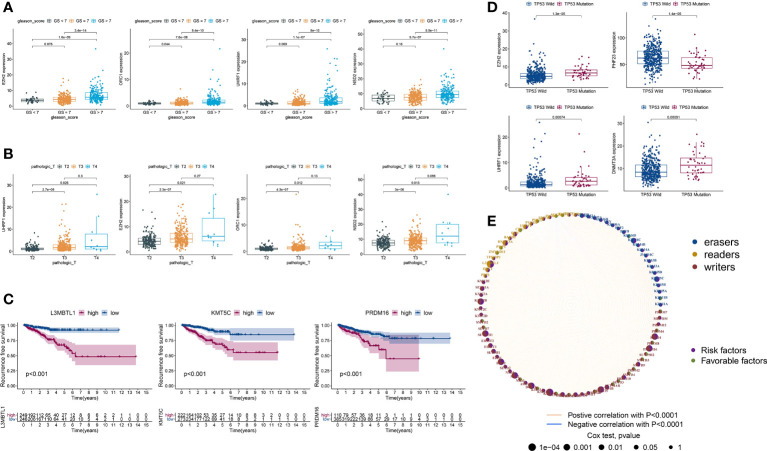
DEGs of HLM regulators in relation to different PCa clinicopathological characteristics and prognoses. **(A–D)** Distribution of the 3–4 lowest P values of HLM regulators in TCGA-PRAD data stratified by GS (GS > 7 vs. GS < 7) **(A)**, pT (T3 vs. T2) **(B)**, RFS (lowest log-rank P value in the Kaplan‒Meier analysis) **(C)**, and TP53 (mutation vs. wild type) **(D)**. The boxplots show the median ± interquartile range values, and P values are presented above each pair of comparisons. **(E)** Prognostic network of interactions among HLM regulators in PCa. Different circle sizes represent the P values of each HLM regulator with respect to the prognosis. Right hemisphere of purple, risk factors for RFS; green, favorable factors for RFS. Left hemisphere of blue, erasers; orange, readers; red, writers. Lines of pink, positive correlations of HLM regulators; blue, negative correlations.

PCa patients with GS ≥ 7 were previously reported to have more aggressive disease and a worse prognosis than those with GS < 7 (38, 39). Hence, we divided TCGA-PRAD into three groups: 45 with GS < 7; 246 with GS = 7; and 204 with GS > 7. Next, we estimated the expression levels of HLM regulators using TCGA-PARD TPM data. Compared with those in the GS < 7 group, the expression levels of EZH2, ORC1, UHRF1, NSD2, KMT5C, PRDM12, EED, DNMT3A, KDM2A, and KDM2B were significantly elevated in the GS > 7 group (P < 0.001) (**Figure 3A** and **Table S3**). The DEGs in HLM regulators in different pathological T (pT) stages (187 with T2; 291 with T3; and 10 with T4) were also analyzed, and UHRF1, EZH2, ORC1, NSD2, EED, DNMT3A, KMT5C, and PRDM12 were significantly elevated in T3 stage PCa compared with T2 stage PCa (P < 0.001) (**Figure 3B** and **Table S4**).

In the survival analysis of Kaplan–Meier curves and univariate Cox regression analysis, TCGA-PRAD patients were divided into two groups according to the optimum threshold segmentation of the expression levels of HLM regulators, which had the lowest log-rank P value in the Kaplan−Meier analysis. High expression levels of KMT5C, L3MBTL1, PRDM16, NSD2, KMT2B, SUV39H1, SETD4, DOT1L, EZH2, and PRDM15 were associated with poor RFS rates (HR > 1 and P value of Cox regression analysis < 0.001) (**Figure 3C**). PCa with TP53 mutation was previously reported to have poor radiographic progression-free survival (rPFS) rates and a shorter time to CRPC progression (40). We divided TCGA-PRAD into two groups according to TP53 mutation status (44 with TP53 mutation and 429 with TP53 wild-type) and found that the expression levels of EZH2, PHF23, UHRF1, DNMT3A, and SMYD2 were differentially expressed between the TP53 mutation and wild-type groups (P < 0.001) (**Figure 3D** and **Table S5**). The comprehensive landscape of HLM regulator interactions and prognosis based on RFS outcomes is depicted in the network (**Figure 3E**).

**Figure 4:**
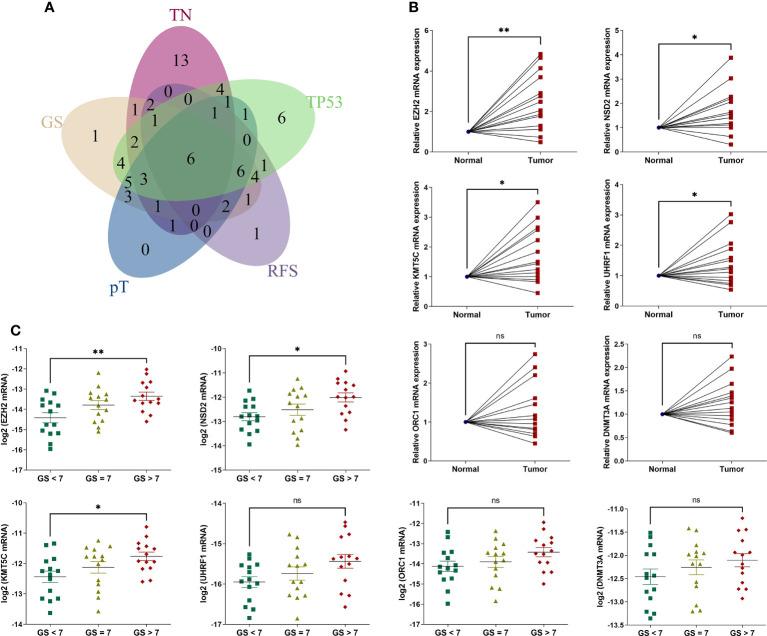
The expression levels of 6 intersecting differentially expressed HLM regulators in clinical PCa tissues. **(A)** The intersecting DEGs of 91 HLM regulators in relation to the comparisons of TN (tumor vs. normal PCa tissues), GS (GS > 7 vs. GS < 7), pT (T3 vs. T2), RFS (P value of univariate Cox regression analysis < 0.05), and TP53 (mutation vs. wild type) are shown as a Venn diagram. **(B, C)** The expression levels of 6 intersecting HLM regulators (EZH2, NSD2, KMT5C, UHRF1, ORC1, and DNMT3A) were compared in PCa vs. adjacent normal prostate tissues **(B)** and GS > 7 vs. GS = 7 vs. GS< 7 **(C)**. The results were normalized to adjacent normal tissues or those of endogenous reference genes. The means ± SEMs are shown in the graphs. ns P > 0.05; *P < 0.05; **P < 0.01.

We then analyzed the intersecting differentially expressed HLM regulators by comparing TN (tumor versus (vs.) normal), GS (GS > 7 vs. GS < 7), pT (T3 vs. T2), RFS (high vs. low in the univariate Cox regression analysis), and TP53 (mutation vs. wild type) (P < 0.05). Six intersecting DEGs of EZH2, NSD2, KMT5C, UHRF1, ORC1, and DNMT3A were found in the 5 comparisons, and all were consistently highly expressed in tumor and other advanced-stage parameters (in the GS > 7, pT3, HR > 1, and TP53 mutation groups) (**Figure 4A**). Forty-two clinical PCa tissues were used to validate these oncogenes, including 14 adjacent normal prostate tissues, 14 with GS < 7, 14 with GS = 7, and 14 with GS > 7 (**Table 1**). The expression levels of 6 genes were assessed *via* qPCR analysis. Compared with adjacent normal tissues, the relative expression levels of EZH2, NSD2, KMT5C, and UHRF1 were higher in PCa tissues (P < 0.05), but no significant differences were found in the expression levels of ORC1 and DNMT3A (P > 0.05) (**Figure 4B**). The expression levels of EZH2, NSD2, and KMT5C were significantly higher in the GS > 7 group than in the GS < 7 group (P < 0.05) (**Figure 4C**). These data confirmed the roles of EZH2, NSD2, and KMT5C oncogenes in the occurrence and progression of PCa.

### Consensus clustering of the PCa cohort *via* HLM regulators

**Figure 5:**
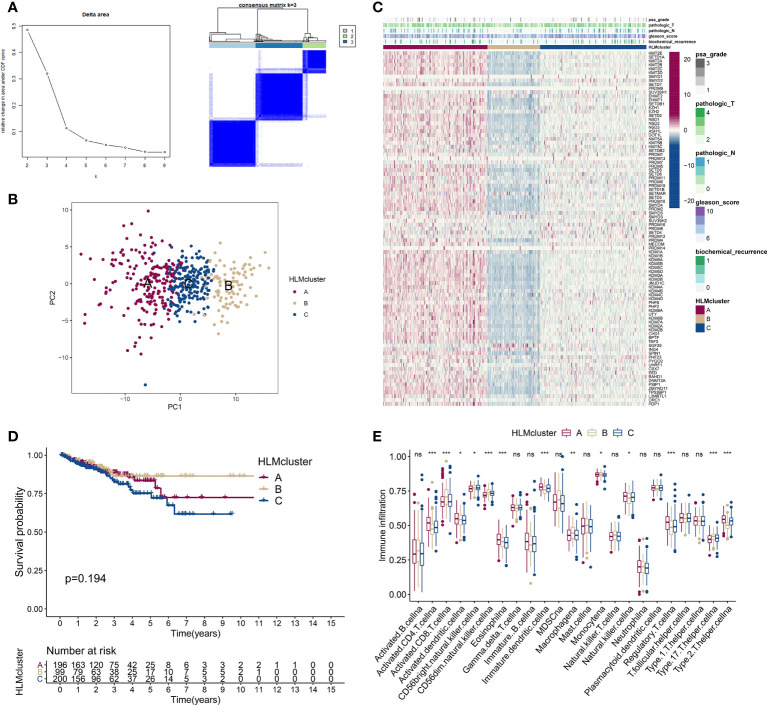
Consensus clustering analysis based on HLM regulators. **(A)** (left) Relative changes in the area under the CDF curve from k = 2 to 9. (right) Color-coded heatmap of the consensus matrix for k = 3 obtained from applying consensus clustering. Color gradients represent values from 0–1: white: 0; dark blue: 1. **(B)** PCA of the transcriptome profiles of HLM regulators in three HLMcluster patterns. Red, orange, and blue dots in the scatter diagram represent HLMclusters (A–C) respectively. **(C)** The expression levels of HLM regulators in the unsupervised three HLMclusters were estimated, and the distribution of the various clinicopathological characteristics of TCGA-PRAD are shown as a heatmap. PSA grade, pT, pN, GS, and biochemical recurrence were used for patient annotation. Red in the heatmap, high expression; blue, low expression. **(D)** RFS analysis among the three HLMclusters using Kaplan–Meier curves. Log-rank P values are shown in the graph, and the numbers at risk are shown at the bottom. **(E)** The abundance of infiltration of each immune cell type among the three HLMclusters is shown in the boxplot. The values of the median ± interquartile range are shown in the graph. ns P > 0.05; *P < 0.05; **P < 0.01; ***P < 0.001.

We divided TCGA-PRAD patients into subgroups (HLMcluster) through consensus clustering analysis using HLM regulators to explore the influence of HLM modification patterns on PCa prognosis and immune cell infiltration. The cluster number, k = 3, was determined to be the optimal category number considering the appreciable delta area under the CDF curves (**Figure 5A**). The transcriptome profiles of HLM regulators between different clusters (named “clusters 1–3 or A-C”) were significantly distinguished by PCA (**Figure 5B**). The levels of HLM regulators were integrally highly expressed in cluster A and weakly expressed in cluster B (**Figure 5C**). Among the three clusters, the number of biochemical recurrences, pathological N1 (pN1), and PSA grade 3 (grade 1: > 0 and < 1, grade 2: 1–10, grade 3: > 10) stages were visually lowest in cluster B, implying relatively low malignancy of PCa in cluster B (**Figure 5C**). The different KEGG pathways were analyzed *via* GSVA in every pairwise comparison among the three clusters (**Figure S2A-C**). The KEGG lysine degradation, adherens junction, and neurotrophin signaling pathways were dysregulated in the comparison of clusters A and B (**Figure S2A**). However, the RFS Kaplan–Meier curves for the HLMclusters revealed no significant differences among these subgroups (P = 0.194) (**Figure 5D**). Thus, HLM clusters may not be suitable for prognostic risk prediction. Immune cells can infiltrate more oncogenic mutated tumors, and these tumors are more sensitive to immunotherapy (41, 42). We analyzed immune cell infiltrations in three HLMclusters and found that 13 of 23 subpopulations of immune cells were significantly differentially infiltrated among these clusters (**Figure 5E** and **Table S6**).

### Consensus clustering of the PCa cohort *via* DEGs in HLMclusters

**Figure 6:**
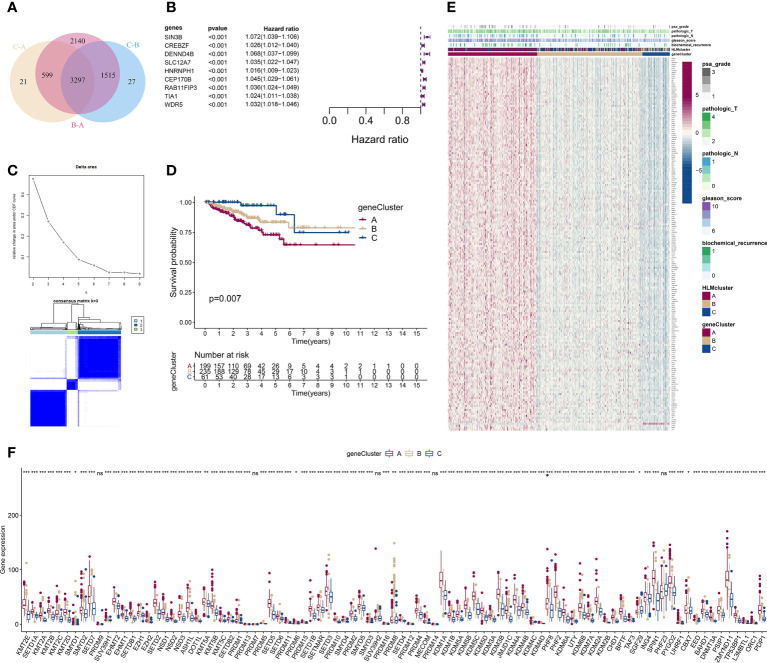
Consensus clustering analysis based on 189 HLM phenotype-related genes. **(A)** Intersecting DEGs identified in every pairwise comparison of HLMclusters are shown as a Venn diagram. **(B)** Univariate Cox regression analysis for RFS was performed in relation to 3,297 intersecting genes. The statistical significance (P in the Kaplan–Meier survival and Cox regression analysis < 0.001) of 9 genes (SIN3B, CREBZF, DENND4B, SLC12A7, HNRNPH1, CEP170B, RAB11FIP3, TIA1, and WDR5), P value, and HR value (with 95% confidence interval (CI)) are shown in the plot. **(C)** Consensus clustering analysis based on the 189 HLM phenotype-related genes (overlap in the pairwise comparisons of HLMclusters and P < 0.01 in the Cox regression analysis) was performed. (up) Relative change in area under the CDF curve from k = 2 to 9. (down) Color-coded heatmap of the consensus matrix for k = 3. Color gradients represent values from 0–1: white: 0; dark blue: 1. **(D)** RFS analysis among the three geneClusters using Kaplan–Meier curves. Log-rank P values are shown in the graph, and the numbers of at-risk patients are shown at the bottom. **(E)** The expression levels of 189 HLM phenotype-related genes in the unsupervised three geneClusters were estimated, and the distribution of the various clinicopathological characteristics of TCGA-PRAD are shown as a heatmap. PSA grade, pT, pN, GS, biochemical recurrence, and HLMclusters were used for patient annotation. Red in the heatmap, high expression; blue, low expression. **(F)** Boxplot of the expression levels of HLM regulators among the three geneClusters. The values of the median ± interquartile range are shown in the graph. ns P > 0.05; * P < 0.05; ** P < 0.01; ***P < 0.001.

We screened DEGs (P value of Bayes test < 0.00001) in every pairwise comparison among the three HLMclusters to investigate the effect of downstream genes of HLM regulators and found 3,297 intersecting genes *via* a Venn diagram (**Figure 6A**). In the GO enrichment analysis, epigenetic reprogramming, such as covalent chromatin modification, histone modification, RNA splicing, nuclear speck, and transcription coregulator activities, was significantly enriched according to the DEGs (**Figure S3A, B**). KEGG pathway signaling enrichment analysis of the DEGs revealed that nucleocytoplasmic transport, protein processing in the endoplasmic reticulum, ubiquitin-mediated proteolysis, and mRNA surveillance pathways were dysregulated (**Figure S3C, D**).

We then performed a univariate Cox analysis using these DEGs and found that 189 genes (HLM phenotype-related genes) had significant differences in the context of RFS outcomes (P value of Cox regression< 0.01). Nine genes (SIN3B, CREBZF, DENND4B, SLC12A7, HNRNPH1, CEP170B, RAB11FIP3, TIA1, and WDR5) among these had a P value < 0.001 in the Kaplan–Meier survival and Cox regression analysis (**Figure 6B**).

We next evaluated the characteristics of classify patterns of HLM phenotype-related genes. Three consensus clusters (geneCluster 1–3 or A–C) of TCGA-PRAD patients were identified by analyzing 189 HLM phenotype-related genes (**Figure 6C**). In the survival analysis, significant differences in the RFS outcomes were found among geneClusters, and cluster A had the poorest RFS rate (P value of Kaplan–Meier survival analysis = 0.007) (**Figure 6D**). The distribution of clinicopathological characteristics in the geneClusters showed that the number of biochemical recurrences, GS, pN, pT, and PSA grade 3 were visually highest in geneCluster A (**Figure 6E**). The TME of immune cell infiltration characteristics in the three geneClusters were evaluated, and 7 of 23 subpopulations of immune cells were differentially infiltrated among these clusters (**Figure S4A** and **Table S7**). The expression levels of HLM regulators in the geneClusters revealed that 86 of 91 HLM regulators were significantly dysregulated, most of which were highly expressed in cluster A (**Figure 6F** and **Table S8**).

### A low HLMscore is associated with poor RFS outcomes

**Figure 7:**
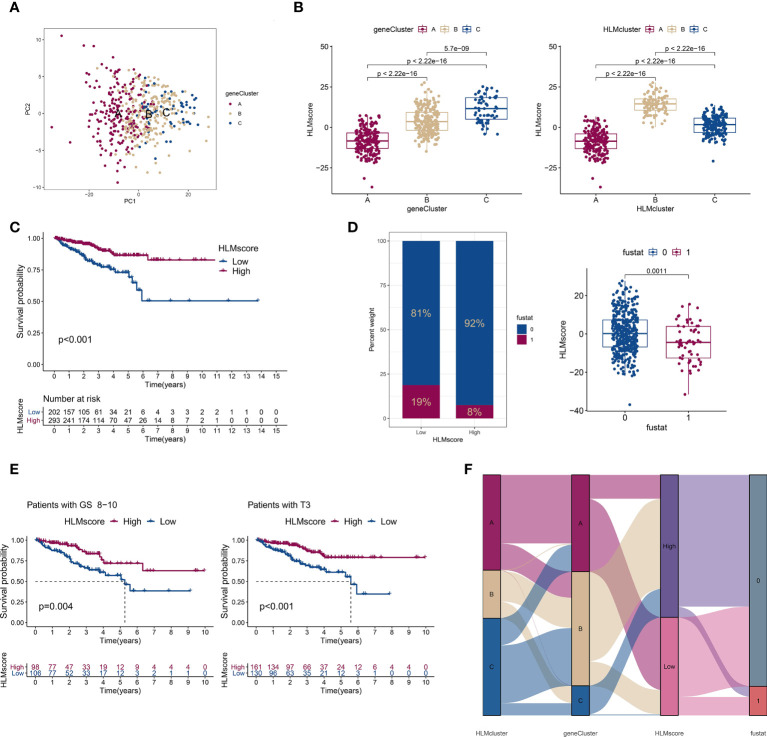
The low-HLMscore group based on the 189 HLM phenotype-related genes was associated with poor prognosis in terms of RFS. **(A)** PCA of the transcriptome profiles of 189 HLM phenotype-related genes in three geneGluster patterns. Red, orange, and blue dots in the scatter diagram represent geneClusters A to C, respectively. **(B)** The HLMscore was calculated *via* PCA based on 189 gene levels in TCGA-PARD. Then, we compared the levels of the HLMscore among geneClusters (left) and HLMclusters (right). The values of median ± interquartile range and P are shown in the boxplots. **(C)** TCGA-PRAD patients were divided into two groups (202 with low-HLMscore group and 293 with high-HLMscore group) according to the optimum threshold segmentation of the HLMscore in relation to the lowest log-rank P value in the Kaplan‒Meier analysis. RFS analysis of the two HLMscore groups is performed using Kaplan–Meier curves. **(D)** (left) The proportion among TCGA-PRAD patients of biochemical recurrence status in low- and high-HLMscore groups (fustat of 0: no recurrence; 1: recurrence). (right) HLMscores in the different statuses of biochemical recurrence. The P value of the Wilcoxon test is shown in the boxplot. **(E)** RFS analysis of the two HLMscore groups among TCGA-PRAD patients with GS 8–10 (left) and pT3 (right). **(F)** The attribute changes of TCGA-PRAD in HLMclusters, geneClusters, HLMscore, and recurrence status are shown as an alluvial diagram.

Considering the complexity of HLM regulators and the individual heterogeneity of PCa patients, the patterns of HLM modification in individual patients were quantitatively assessed (HLMscore) by constructing a set of scoring system by PCA of the 189 HLM phenotype-related genes (**Figure 7A**). The HLMscores in geneClusters/HLMclusters were analyzed and were significantly different in both clusters; geneCluster C and HLMcluster B had the highest values (**Figure 7B**). In the survival analysis, TCGA-PRAD patients were divided into two groups according to the optimum threshold segmentation of the HLMscore with the lowest log-rank P value in the Kaplan−Meier analysis (202 with low-HLMscore group and 293 with high-HLMscore group). The results showed that the low-HLMscore group was associated with poor RFS outcomes (P < 0.001) (**Figure 7C**). A low HLMscore was also associated with a higher rate of biological recurrence (fustat of 1) (19%), and the recurrence status was matched with lower HLMscores (**Figure 7D**). We then analyzed the RFS outcomes of the HLMscore groups stratified by clinicopathological features of GS and pT (**Figure 7E** and **Figure S4B, C**). The low HLMscore group also exhibited poor RFS outcomes in the GS 8-10 (P = 0.004) and pT3 (P < 0.001) stages (**Figure 7E**). A summary of the attribute changes in HLMcluster, geneCluster, HLMscore, and biological recurrence status is shown in the alluvial diagram (**Figure 7F**). Consistent with the findings above, most patients in geneCluster A were classified into the low-HLMscore group, which is relevant to the higher rate of biological recurrence.

### Characteristics of gene mutations based on HLMscore status in TCGA-PRAD

**Figure 8:**
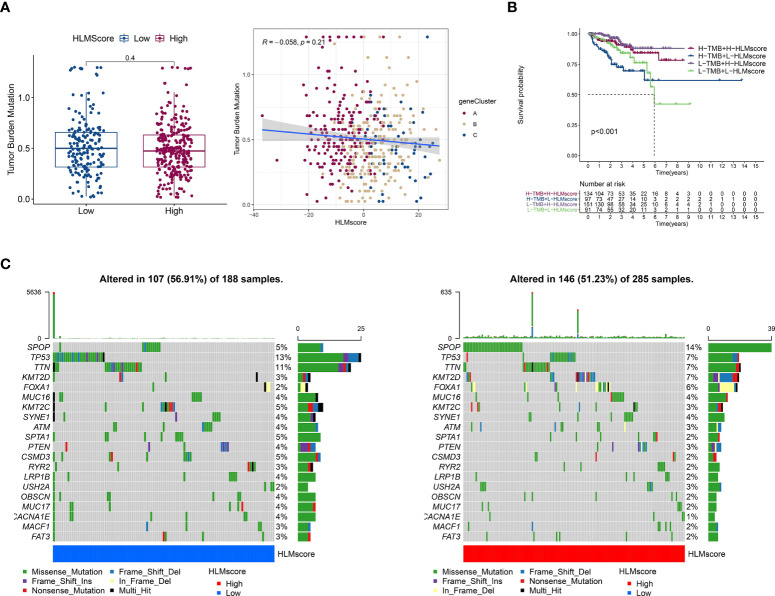
Characteristics associated with TCGA-PRAD tumor mutations based on HLMscore status. **(A)** (left) TMB values of the two HLMscore groups. The P value of the Wilcoxon test is shown in the boxplot. (right) Spearman correlation analysis between the HLMscores and TMB values in TCGA-PRAD was performed and is shown as a scatter diagram. Red, orange, and blue dots represent geneClusters A to C, respectively. **(B)** TCGA-PRAD patients were divided into two statuses (low and high TMB) according to the optimum threshold segmentation of TMB values in relation to the RFS analysis. Then, the TCGA-PRAD patients were further split into four groups based on TMB and HLMscore status (high (H)-TMB + H-HLMscore; H-TMB + low (L)-HLMscore; L-TMB + H-HLMscore; and L-TMB + L-HLMscore), and survival analysis for RFS was performed. **(C)** The somatic mutations of TCGA-PRAD patients with low-HLMscore (left) and high-HLMscore (right) groups are shown as waterfall plots.

TMB is positively associated with oncogenic mutations and the immunotherapeutic response (41, 42). The correlation between the HLMscore and TMB was analyzed to estimate PCa malignancy. However, the results showed that there was no difference in the level of TMB between the low- and high-HLMscore groups (P = 0.4), and TMB also showed no correlation with the HLMscore in the Spearman correlation analysis (R = -0.058 and P = 0.21) (**Figure 8A**). The patients were first divided into two groups (the high- and low-TMB groups) according to the TMB value *via* an RFS analysis as previously reported (29). Then, the patients were further divided into four groups by combining the HLMscore and TMB groups. As shown in **Figure 8B**, the high-TMB with low-HLMscore group had the poorest RFS among the four groups. The distribution of somatic mutations in the low- and high-HLMscore groups indicated a more extensive mutation frequency of TP53 (13% vs. 7%) and a reduced frequency of SPOP (5% vs. 14%) mutation in the low-HLMscore group vs. the high-HLMscore group (**Figure 8C**).

### Characteristics of immune cell infiltration based on the HLMscore in TCGA-PRAD

**Figure 9:**
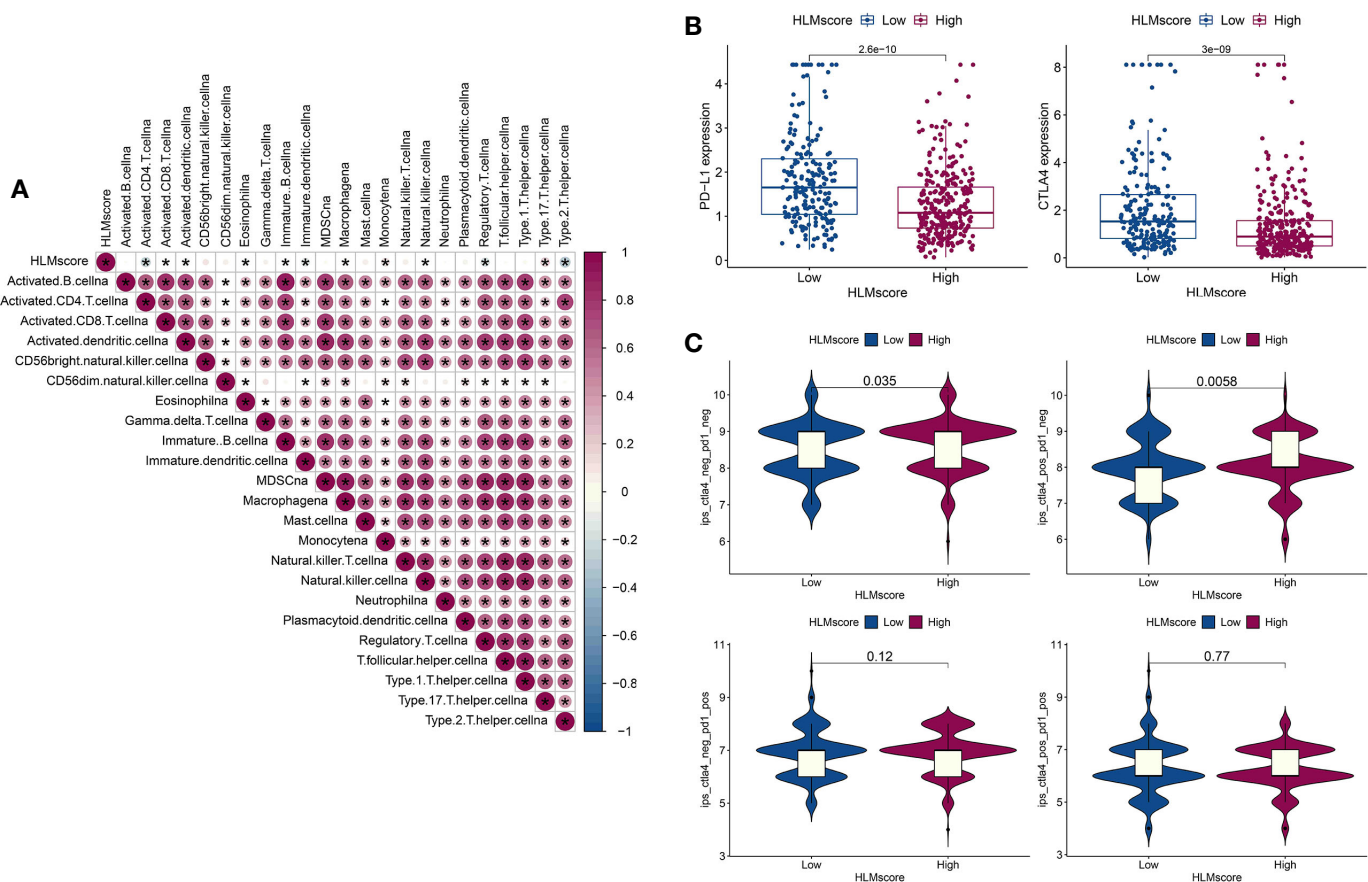
Characteristics of the HLMscore status in PCa with an immune-related microenvironment. **(A)** Correlation analysis between the HLMscore and infiltrating immune cell type in TCGA-PRAD. Red, positive correlation, blue, negative correlation.*P < 0.05. **(B)** The expression levels of PD-L1 and CTLA4 in the two HLMscore groups are shown as boxplots. **(C)** The IPS scores in ips_ctla4_neg_pd1_neg (CTLA4 and PD1 negative response), ips_ctla4_pos_pd1_neg (CTLA4 positive and PD1 negative response), ips_ctla4_neg_pd1_pos, and ips_ctla4_pos_pd1_pos in relation to the respective HLMscore groups are shown as violin plots. The P values of the Wilcoxon test are shown in the violin plots.

The correlation between the HLMscore and genes related to immune-related cell infiltration was analyzed to better illustrate the HLMscore characteristics based on immune cell infiltration. Previous research suggested that low immune-cell infiltration was markedly related to prolonged overall survival times (30). Our results revealed that 8 of 23 immune cell infiltrates were negatively correlated with the HLMscore, and 4 were positively correlated (**Figure 9A**). Thus, it is unlikely that the HLMscore and immune response patterns interact with each other to influence RFS prognosis (**Figure 7C**).

Immunotherapy as represented by immunological checkpoint (PD-1/L1 and CTLA-4) blockade (ICB) was confirmed to have pronounced efficacy in the clinical treatment of durable response patients (43). However, unfortunately, the majority of tumor patients are insensitive and experience minimal or no clinical benefit, which is far from ideal (43). Predicting sensitivity to ICB is key for improving ICB therapeutic success and exploring novel immunotherapeutic strategies (42, 44). PD-L1 and CTLA4 expression in the HLMscore groups was evaluated to determine the potential sensitivity to immunotherapy. The results showed that both PD-L1 and CTLA4 were highly expressed in the low-HLMscore group (**Figure 9B**). We then performed value prediction of the risk score for ICB. TCGA-PRAD patients were classified into the following four groups: ips_ctla4_neg_pd1_neg (CTLA4 and PD1 negative response); ips_ctla4_pos_pd1_neg (CTLA4 positive and PD1 negative response); ips_ctla4_neg_pd1_pos; and ips_ctla4_pos_pd1_pos (**Figure 9C**). The results showed that CTLA4-positive and -negative responses accompanied by PD1-negative regions (ips_ctla4_pos/neg_pd1_neg) had significantly different IPSs in the two HLMscore groups (P < 0.05) but not in the PD1-positive regions (ips_ctla4_pos/neg_pd1_pos) (P > 0.05) (**Figure 9C**). This finding suggests that the HLMscore groups in this risk score model may indicate a better prediction efficacy to predict insensitivity to PD1 blockade.

## Discussion

In this study, we performed bioinformatic analysis to evaluate the extensive regulatory mechanism of the HLM modification in PCa for the first time using the TCGA-PRAD dataset. We found that HLM modification patterns play a crucial role in PCa aggressiveness, prognosis, and prediction of immunotherapy sensitivity. Three representative HLM regulators (EZH2, NSD2, and KMT5C) were found to serve as highly valuable biomarkers in advanced PCa.

In general, the molecular mechanism of PCa progression remains unclear. Increasing evidence has demonstrated that epigenetic reprogramming through HLM can accelerate the development of PCa (14, 15). Epigenetic regulation by the degree of HLM occurs through alterations in gene transcription, chromatin structure, and mitosis (45). The most acknowledged HLM regulator is EZH2, a subunit of polycomb repressive complex 2 (PRC2) that silences gene expression *via* H3K37me3 methyltransferase activity and has been reported to be associated with the progression of CRPC and NEPC (16–19, 46). Unlike its role as a transcriptional repressor, the phosphorylation of EZH2 is associated with both coactivation (16, 47, 48) and corepression (15, 18) of AR transcriptional activity in PCa. Previous patient studies confirmed the value of EZH2 as a PCa prognosis parameter before (20) or after (21) treatment.

Moreover, as an H3K4 methyltransferase, SMYD3 can epigenetically upregulate AR expression by binding to the AR promoter region and is further associated with PCa tumorigenesis (25). R. Vatapalli *et al.* reported that the H3K79 methyltransferase DOT1L selectively regulates tumorigenicity and is associated with poor PCa outcomes by coordinating with AR and regulating MYC transcription (26). LSD1 acts as a transcriptional repressor by associating with FOXA1 through the demethylation of H3K4; it also acts as an AR coactivator, and an LSD1 inhibitor suppresses tumor growth synergy with enzalutamide in CRPC cells (22). NSD2 is a conserved driver of metastatic PCa progression, which robustly expressed in lethal PCa and its silencing inhibited PCa metastasis *in vivo* of mouse allografts (24). Our previous study also revealed that MLL5α inhibits PCa progression by forming a complex with AR and promotes the transcription of NDRG1 through H3K4me3 in the promoter region (28). All of these previous studies suggest that epigenetic regulation *via* HLM regulators plays a crucial role in PCa progression.

Nevertheless, the overall effect of HLM modification in PCa remains unclear. To our knowledge, only one preliminary study has been conducted to analyze the genetic abnormalities of HMT in TCGA-PRAD, but this study lacked integration with HDM and MLRP (13). The complexity and heterogeneity of the individual PCa microenvironment may be influenced by various HLM regulators that interact with each other and form a complex network to induce epigenetic reprogramming. Thus, we summarized the widely acknowledged HLM regulators, 51 writers (HMT), 21 erasers (HDM), and 19 readers (MLRP), and then comprehensively investigated the HLM modification patterns in PCa by assessing various clinicopathological characteristics and performing survival analysis using the TCGA-PRAD dataset.

The results revealed that more than half (51 of 91) of the HLM regulators are dysregulated in PCa, and the numbers of overexpressed and downregulated HLM regulators were approximately the same (26 vs. 25), suggesting various alterations of HLM regulators in PCa. The representative HLM regulator EZH2 was overexpressed, but negligible CNV was found in PCa. All HLM regulators showed a copy number gain of < 5%. The highest copy number loss (25.5%) was found in SETDB2, which was downregulated in PCa patients (P = 0.02). However, while HLM regulators, such as PRDM13, PRDM1, KDM6B, CHD1, PHF23, and PRDM7, all exhibited high levels of copy number loss (> 10%), no significant downregulation (even overexpression of PRDM13) was found in the PCa of TCGA-PRAD dataset. This finding implies no causal relationship between CNV and the expression levels of HLM regulators in PCa.

In the prognosis validation by survival analysis, we conducted RFS instead of OS because of the low death rate (10 of 493) among TCGA-PRAD patients. We extracted the follow-up phenotype of “days_to_first_biochemical_recurrence” as “recurrence follow-up time” and confirmed “recurrence status”, as previously described (29). The Cox regression analysis showed that 10 HLM regulators (KMT5C, L3MBTL1, PRDM16, NSD2, KMT2B, SUV39H1, SETD4, DOT1L, EZH2, and PRDM15) were significant risk factors, with HR > 1 and P < 0.001.

We validated the oncogene status of HLM regulators *via* integration with DEGs associated with various clinicopathological characteristics (TN, GS, pT, and TP53 mutation) and RFS outcomes and found 6 intersecting genes, EZH2, NSD2, KMT5C, UHRF1, ORC1, and DNMT3A, which were highly expressed in association with all advanced-stage parameters (tumor, GS > 7, pT3, HR > 1, and TP53 mutation). When verifying these genes in clinical PCa tissues, six genes trended toward an increase in the GS > 7 group (compared with GS < 7), and 3 of them (EZH2, NSD2, and KMT5C) were statistically significant (P < 0.05). EZH2 and NSD2 are representative HLM regulators that were mentioned above and have been widely confirmed in PCa research. However, there is a lack of research focused on the role of KMT5C in PCa. KMT5C is an H4K20me3 methyltransferase and represses several key drivers of the epithelial state, further promoting epithelial to mesenchymal transition (EMT) in pancreatic cancer (49). UHRF1 promotes CRPC progression by triggering AR-regulated CDC6 transcription by binding to the CCAAT motif and recruiting KDM4C to demethylate H3K9me2/3 (50). UHRF1 could induce epigenetic inactivation of tumor suppressor genes in combination with SUV39H1, DNA methyltransferases, and EZH2 (51). ORC1 is a key subunit of the origin recognition complex, and few studies have focused on the role of ORC1 in PCa. DNMT3A epigenetically regulates EMT-associated key microRNAs to promote PCa metastasis (52); it is recruited to the promoters of these miRNAs and silences their transcription by increasing H3K9/27me3 and/or decreasing H3K4/36me3. In general, clinical PCa verification and previous research confirmed a certain level of accuracy of our bioinformatic analysis using the TCGA-PRAD dataset.

This study aimed to evaluate the integral HLM phenotypes in PCa patients. Here, we revealed three distinct HLM modification patterns based on 91 HLM regulators (HLMclusters) using consensus clustering analysis and investigated the effect of three HLMclusters on clinicopathological features and the prognosis of PCa patients; the results indicated no significant difference in these clusters in RFS (P = 0.194). This finding implies that clustering TCGA-PRAD patients based on 91 HLM regulators may not be suitable for involvement in a prognostic risk prediction model. We previously divided TCGA-PRAD patients into three clusters based on 25 N6-methyladenosine (m6A) regulators and found no significant differences in RFS analysis (29), which is discordant with the same analysis in gastric cancer (31). This finding suggests the heterogeneity of epigenetic regulation in diverse tumors.

Epigenetic modification of target genes to regulate their transcription is the primary function of HLM regulators. To predict genes downstream of HLM regulators, we identified 189 HLM phenotype-related genes that overlapped in the pairwise comparisons of HLMclusters (P value of Bayes test < 0.00001) and P < 0.01 in the Cox regression analysis. The patients were then classified into three subtypes (geneCluster) based on 189 HLM phenotype-related genes. These subtypes were significantly associated with different RFS rates (P = 0.007), suggesting that it may be an appropriate risk prediction model. In the results, geneCluster A exhibited the poorest RFS and recruited more of the clinicopathological parameters of PSA grade 3, pT4, pN1, and GS > 7.

However, the evaluation above was a qualitative analysis that highly depended on the population of patients and could not accurately predict the HLM modifications in individual PCa patients. Considering the heterogeneity of individual patients and complexity of HLM modifications, we constructed a set of scoring system (HLMscore) as described previously (31) based on the PCA of the 189 HLM phenotype-related genes to quantify the HLM modification pattern of individual PCa patients. The HLMscore progressively increased from geneCluster A–C, and a lower HLMscore resulted in a poorer prognosis in terms of RFS. The TMB score corresponds to tumor-related mutations that are sensitive to immune cell recruitment (41, 42). This study found no significant correlation between TMB and HLMscore (P = 0.21). However, the low-HLMscore group exhibited a higher proportion of TP53 mutations (13% vs. 7%) and fewer SPOP mutations (5% vs. 14%) than the high-HLMscore group. TP53 and SPOP were the most highly mutated genes in PCa (11% and 10%, respectively, in TCGA-PRAD). TP53 mutation was associated with a poor rPFS outcome, shorter time to CRPC progression, and higher aggressiveness (40, 53). SPOP mutation can enhance autophagy in PCa and respond to AR inhibition in various clinical settings (54, 55). Epigenetic modulation has been reported to play a vital role in antitumor immunity (56), but few studies have focused on HLM regulators and tumor immune cell infiltration. We found that 12 of 23 immune cell infiltrations were significantly correlated with HLMscore, and 8 of them were negatively correlated. The difference in the infiltrated subpopulations of immune-associated cells was also not substantial among the three HLMclusters (13 of 23) and three geneClusters (7 of 23), making it difficult to assess whether the HLM signature influences immune response patterns in PCa.

Immunotherapy with ICB (PD-1/L1 and CTLA-4) was demonstrated to have astounding efficacy in the positive response patients (43). Predicting the sensitivity to ICB in PCa patients is crucial for improving ICB therapy success and exploring novel immunotherapeutic strategies (42, 44). We found significant differences in the expression levels of PD-L1 and CTLA4 in the low- and high-HLMscore groups. Further assessment of immunotherapy responses showed that the IPS of CTLA4-positive and -negative responses accompanied by a PD1-negative response significantly differed between the two HLMscore groups (P < 0.05). This finding suggests that HLMscore estimation may have predictive value for immunotherapy sensitivity in PCa.

## Conclusions

This study comprehensively evaluated the extensive HLM regulation mechanisms in the PCa microenvironment for the first time using the TCGA-PRAD dataset. HLM modification patterns play a crucial role in the complexity and heterogeneity of individual tumor microenvironments. Elucidating the overall effect of HLM regulators in PCa may contribute to the determination of a valuable risk model for predicting prognosis, aggressiveness, and immunotherapy strategies. We found 3 crucial HLM regulators (EZH2, NSD2, and KMT5C) that were consistently highly expressed in advanced PCa stages and associated with various clinicopathological characteristics (tumor, GS > 7, pT3, HR > 1, and TP53 mutation).

## Data availability statement

The datasets presented in this study can be found in online repositories. The names of the repository/repositories and accession number(s) can be found in the article/**Supplementary Material**.

## Ethics statement

Human prostate samples were provided by Beijing Tongren Hospital and Beijing Chaoyang Hospital. Ethical consent was approved by the Ethics Committee of Beijing Tongren Hospital and Beijing Chaoyang Hospital, affiliated with Capital Medical University.

## Author contributions

YQ designed the study. YQ and MW performed the qPCR analysis. HP analyzed the data. XZ and HP provided the clinical prostate cancer samples. YQ wrote the manuscript, which was approved by all authors. All authors contributed to the article and approved the submitted version.

## Funding

This study was supported by the National Natural Science Foundation of China (Grant Nos. 82072833 to HP) and the Open Research Fund from Beijing Advanced Innovation Center for Big Data-Based Precision Medicine, Beijing Tongren Hospital, Beihang University & Capital Medical University (Grant No. BHTR-KFJJ-202005 to HP).

## Acknowledgments

We thank Beijing Chaoyang Hospital, Capital Medical University for providing samples from PCa patients.

## Conflict of interest

The authors declare that the research was conducted in the absence of any commercial or financial relationships that could be construed as a potential conflict of interest.

## Publisher’s note

All claims expressed in this article are solely those of the authors and do not necessarily represent those of their affiliated organizations, or those of the publisher, the editors and the reviewers. Any product that may be evaluated in this article, or claim that may be made by its manufacturer, is not guaranteed or endorsed by the publisher.

## Supplementary material

Supplementary Figure 1 CNV variation and mutation frequency of HLM regulators in PCa. **(A)** The location of CNV alterations of HLM regulators on 23 chromosomes. Red dot, copy number gain; blue dot, copy number loss. **(B)** The mutation frequency of HLM regulators in 484 TCGA-PRAD patients is shown in the waterfall plot. Columns represent individual patients, the upper bar plot shows TMB, the right bar plot shows the proportion of each variant type, and the stacked bar plot below shows the transformed fraction of each patient.

Supplementary Figure 2 KEGG enrichment analysis in distinct HLMclusters. **(A-C)** Heatmap of KEGG enrichment analysis, including the activation states of biological pathways, in the respective comparisons of HLMclusters (A vs. B **(A)**, A vs. C **(B)**, and B vs. C **(C)**.

Supplementary Figure 3 Enrichment analysis of 3,297 intersecting DEGs in every pairwise comparison of HLMclusters. **(A, B)** GO enrichment of biological process terms (BP), cellular component terms (CC), and molecular function terms (MF) are shown as a barplot **(A)** and dotplot **(B)**. **(C, D)** KEGG enriched terms in the activation states of biological pathways are shown as a barplot **(C)** and dotplot **(D)**.

Supplementary Figure 4 TME immune cell infiltration in three geneClusters and RFS prognosis based on HLMscore status among TCGA-PRAD patients stratified by various clinicopathological characteristics. **(A)** The abundance of infiltration of each immune cell type among the three geneClusters is shown in the boxplot. The values of the median ± interquartile range are shown in the graph. ns P > 0.05; ** P < 0.01; ***P < 0.001. **(B)** RFS analysis of the two HLMscore groups of TCGA-PRAD patients with GS 6 (left) and GS 7 (right). **(C)** RFS analysis of the two HLMscore groups of TCGA-PRAD patients with pT2 (left) and pT4 (right).

Supplementary Table 1 Oligonucleotide primers of relative genes.

Supplementary Table 2 The expression levels of 91 HLM regulators in TCGA-PRAD and normal tissues. ^a^ ns P > 0.05; * P < 0.05; ** P < 0.01; ***P < 0.001.

Supplementary Table 3 Expression levels of HLM regulators in TCGA-PRAD data stratified by GS. ^a^ ns P > 0.05; * P < 0.05; ** P < 0.01; ***P < 0.001.

Supplementary Table 4 Expression levels of HLM regulators in TCGA-PRAD data stratified by pT stage. ^a^ ns P > 0.05; * P < 0.05; ** P < 0.01; ***P < 0.001.

Supplementary Table 5 Expression levels of HLM regulators in TCGA-PRAD data stratified by TP53 mutation. ^a^ ns P > 0.05; * P< 0.05; ** P< 0.01; ***P< 0.001.

Supplementary Table 6 Abundance of each infiltrating immune cell among the three HLMclusters. ^a^ ns P > 0.05; * P < 0.05; ** P < 0.01; ***P < 0.001.

Supplementary Table 7 Abundance of each infiltrating immune cell among the three geneClusters. ^a^ ns P > 0.05; ** P < 0.01; ***P < 0.001.

Supplementary Table 8 Expression of HLM regulators among the three geneClusters. ^a^ ns P > 0.05; * P < 0.05; ** P < 0.01; ***P < 0.001.

## Abbreviations

PCa, prostate cancer; ADT, androgen-deprivation therapy; CRPC, castration-resistant prostate cancer; HLM, histone lysine methylation; NEPC, neuroendocrine prostate cancer; TCGA, The Cancer Genome Atlas; DEG, differentially expressed gene; TPM, transcripts per kilobase of exome per million; GO, gene ontology; KEGG, Kyoto Encyclopedia of Genes and Genomes; RFS, recurrence-free survival; PCA, principal component analysis; GSVA, gene set variation analysis; GSEA, gene set enrichment analysis; TMB, tumor mutational burden; TME, tumor microenvironment; ssGSEA, single-sample gene set enrichment analysis; IPS, immunophenoscore; GS, Gleason score; qPCR, quantitative real-time PCR; cDNA, complementary DNA; HR, hazard ratio; CNV, copy number variation; CDF, cumulative distribution function; EMT, epithelial to mesenchymal transition; ICB, immune checkpoint blockade; CI, confidence interval; pT, pathological T; pN, pathological N; rPFS, radiographic progression-free survival; pN, pathological N; OS, overall survival.
