# Supplementary material for: Histone lysine methylation patterns in prostate cancer microenvironment infiltration: Integrated bioinformatic analysis and histological validation
Source: Front Oncol. 2022 Sep 28;12:981226. doi: 10.3389/fonc.2022.981226 (PMC9552767; doi:10.3389/fonc.2022.981226)
Supplement: Supplementary Figure 1 — CNV variation and mutation frequency of HLM regulators in PCa. (A) The location of CNV alterations of HLM regulators on 23 chromosomes. Red dot, copy number gain; blue dot, copy number loss. (B) The mutation frequency of HLM regulators in 484 TCGA-PRAD patients is shown in the waterfall plot. Columns represent individual patients, the upper bar plot shows TMB, the right bar plot shows the proportion of each variant type, and the stacked bar plot below shows the transformed fraction of each patient. [file DataSheet_1.docx]

# **Histone lysine methylation patterns in prostate cancer** **microenvironment infiltration: Integrated bioinformatic analysis and histological validation**

## **Running title: Histone lysine methylation regulators in prostate cancer**

Yongjun Quan^1^, Xiaodong Zhang^2^, Mingdong Wang^1^, Hao Ping^1,3,*^

1 Department of Urology, Beijing Tongren Hospital, Capital Medical University, Beijing 100176, China.

2 Department of Urology, Beijing Chaoyang Hospital, Capital Medical University, Beijing 100020, China.

3 Beijing Advanced Innovation Center for Big Data-Based Precision Medicine, Beihang University & Capital Medical University, Beijing Tongren Hospital, Beijing 100176, China.

* Corresponding author: Hao Ping (pinghaotrh@ccmu.edu.cn)

First author: Yongjun Quan (qyongjun@yeah.net)

(A full list of author information is provided in the electronic submission form).

## **Supplementary Figures**


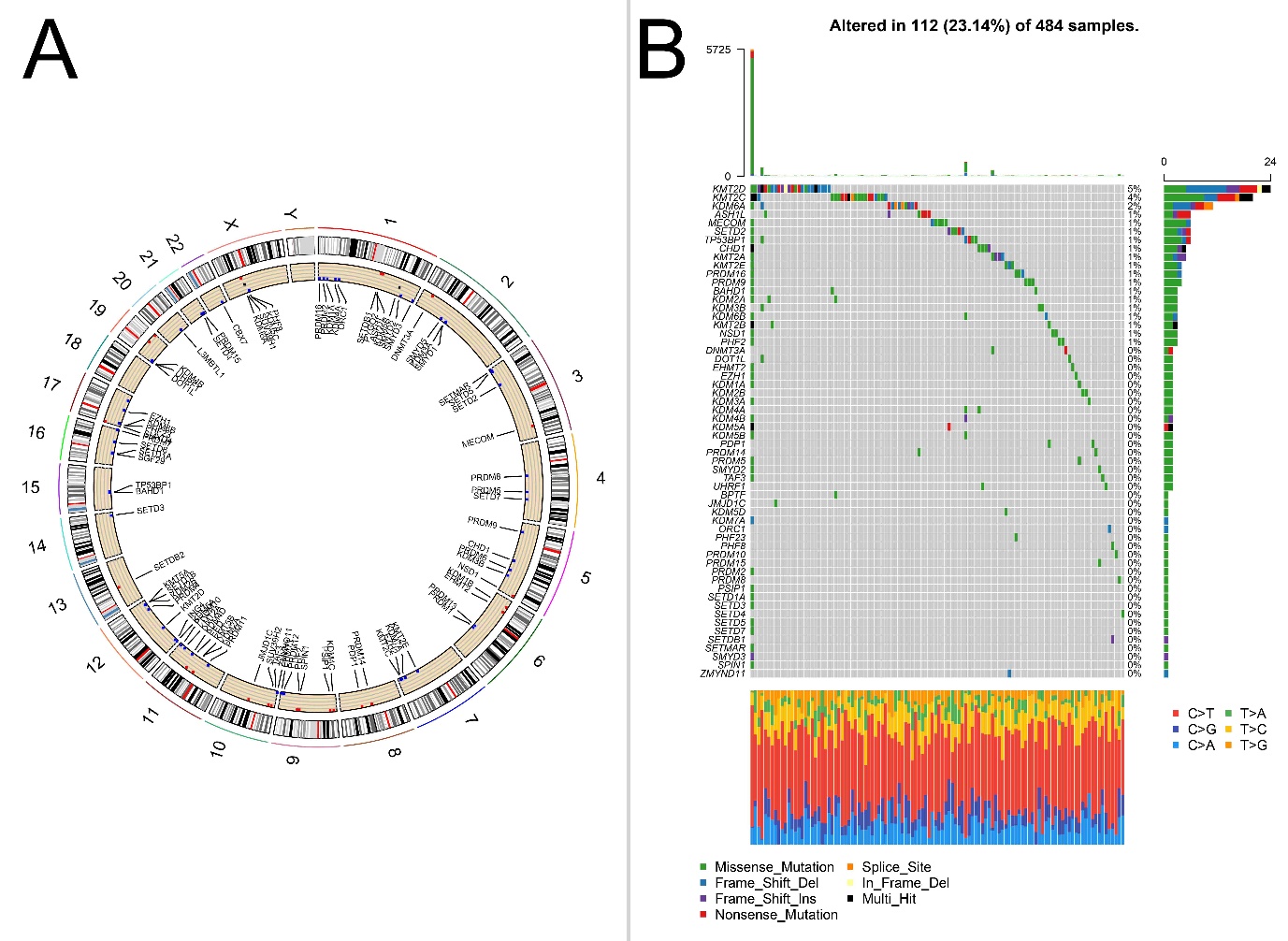


### Fig. S1.

CNV variation and mutation frequency of HLM regulators in PCa. (A) The location of CNV alterations of HLM regulators on 23 chromosomes. Red dot: copy number gain; blue dot: copy number loss. (B) The mutation frequency of HLM regulators in 484 TCGA-PRAD patients is shown in the waterfall plot. Columns represent individual patients, the upper bar plot shows TMB, the right bar plot shows the proportion of each variant type, and the stacked bar plot below shows the transformed fraction of each patient.


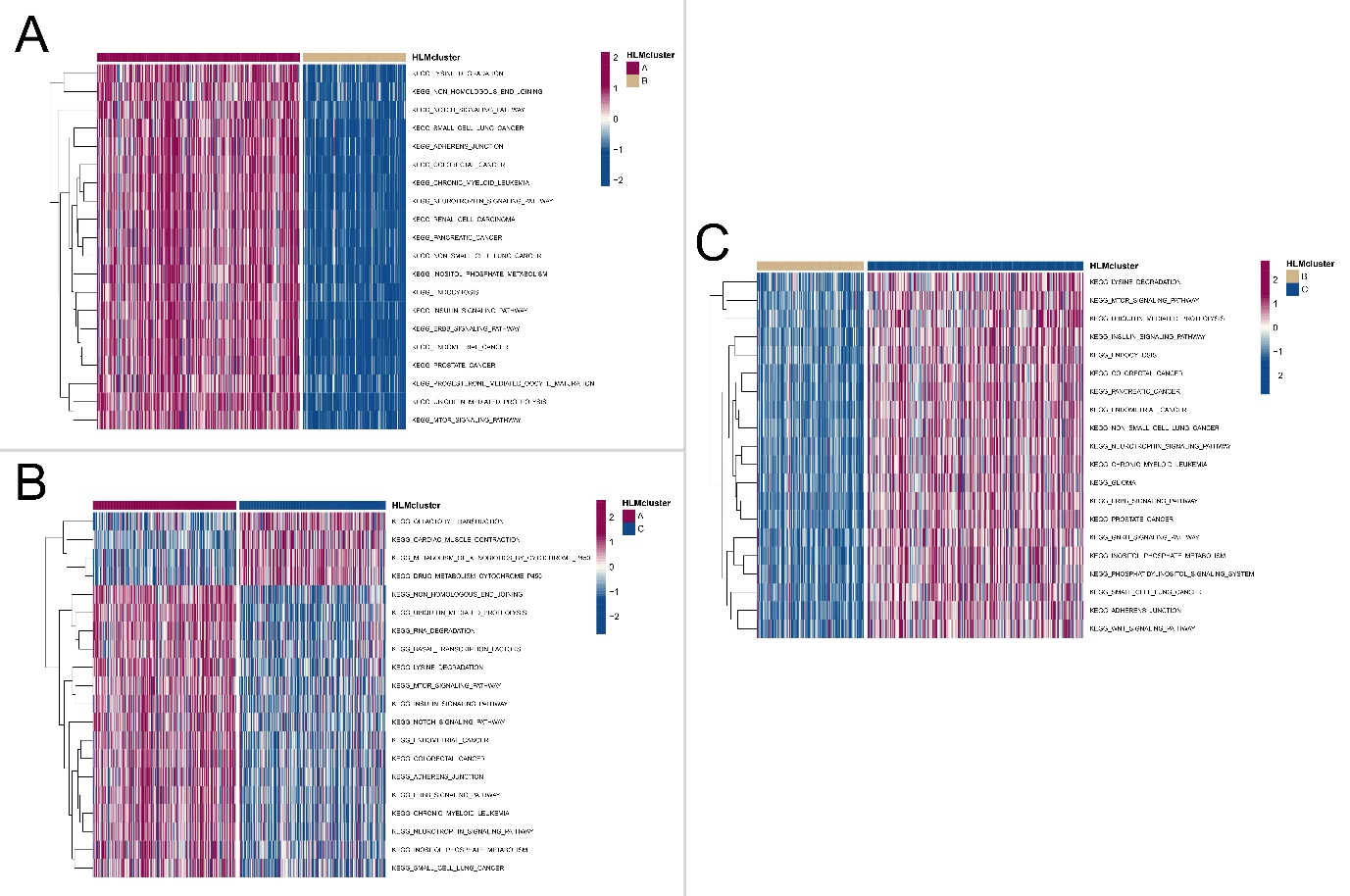


### Fig. S2.

KEGG enrichment analysis in distinct HLMclusters. (A to C) Heatmap of KEGG enrichment analysis, including the activation states of biological pathways, in the respective comparisons of HLMclusters (A vs. B (A), A vs. C (B), and B vs. C (C)).


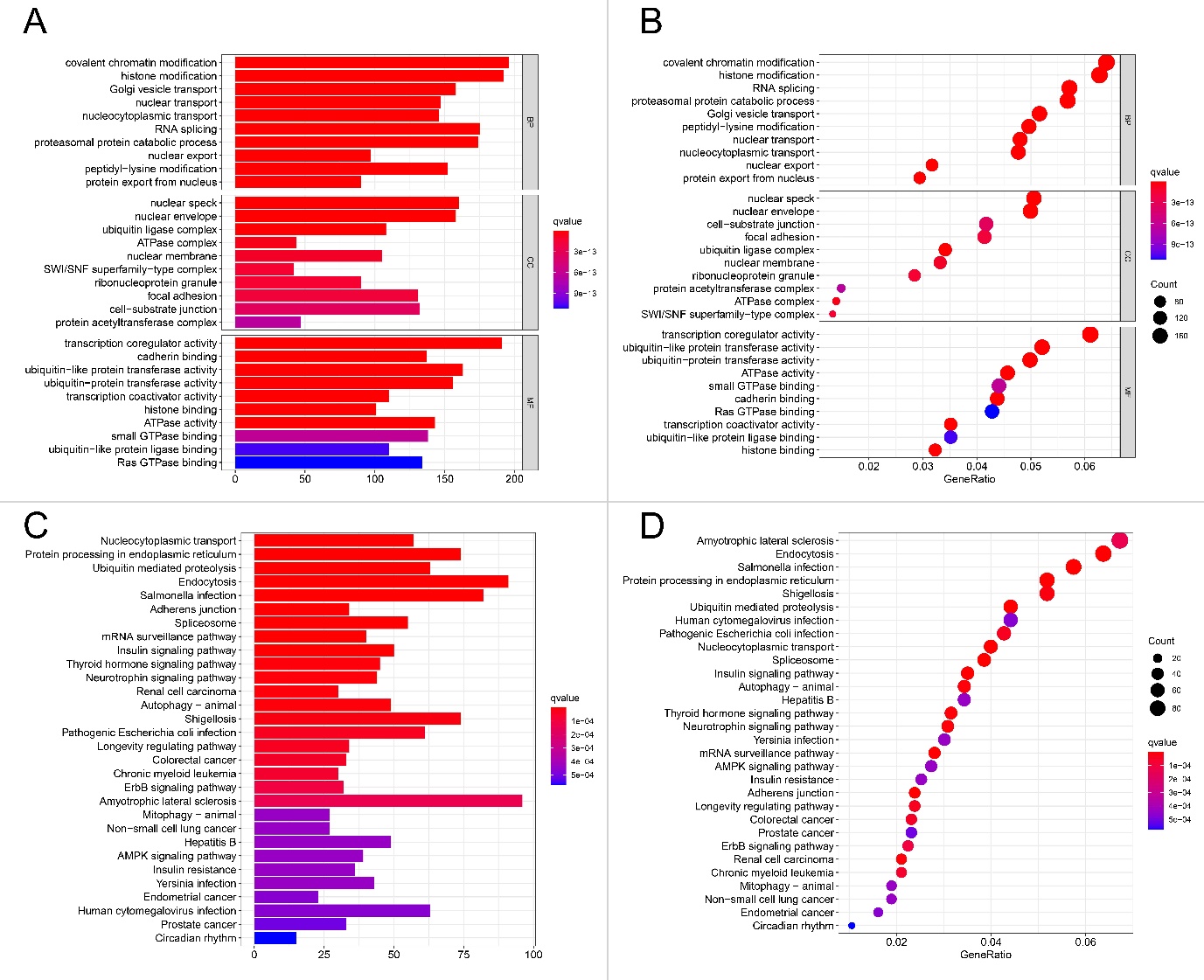


### Fig. S3.

Enrichment analysis of 3,297 intersecting DEGs in every pairwise comparison of HLMclusters. (A and B) GO enrichment of biological process terms (BP), cellular component terms (CC), and molecular function terms (MF) are shown as a barplot (A) and dotplot (B). (C and D) KEGG enriched terms in the activation states of biological pathways are shown as a barplot (C) and dotplot (D).


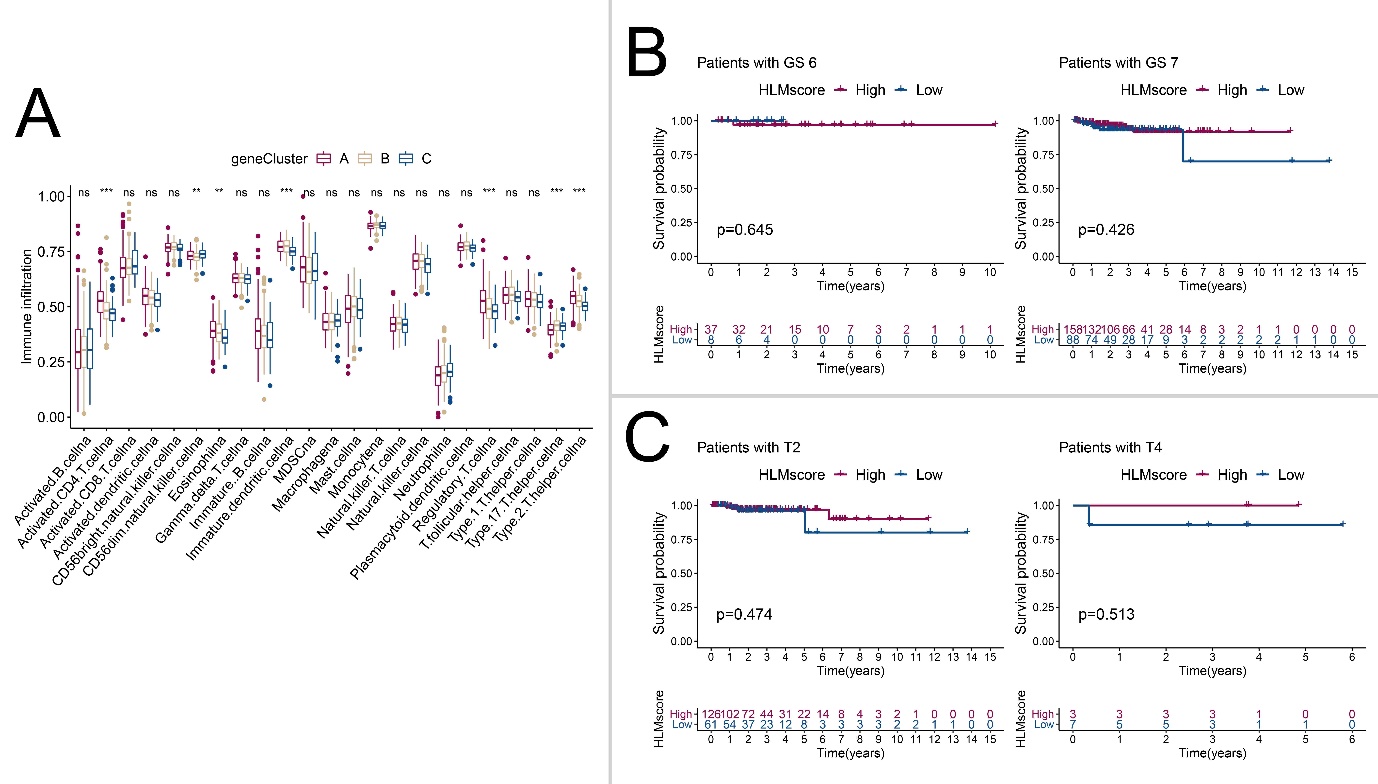


### Fig. S4.

TME immune cell infiltration in three geneClusters and RFS prognosis based on HLMscore status among TCGA-PRAD patients stratified by various clinicopathological characteristics. (A) The abundance of infiltration of each immune cell type among the three geneClusters is shown in the boxplot. The values of the median ± interquartile range are shown in the graph. ns P > 0.05; ** P < 0.01; ***P < 0.001. (B) RFS analysis of the two HLMscore groups of TCGA-PRAD patients with GS 6 (left) and GS 7 (right). (C) RFS analysis of the two HLMscore groups of TCGA-PRAD patients with pT2 (left) and pT4 (right).

## **Supplementary Tables**

### Supplementary Table 1. Oligonucleotide primers of relative genes.

| **Gene** | **Forward** | **Reverse** |
| --- | --- | --- |
| **EZH2** | AATCAGAGTACATGCGACTGAGA | GCTGTATCCTTCGCTGTTTCC |
| **ORC1** | ACTGAGGCTGCATGTTTCTG | CTCCGGTATGGTCAAGGAGT |
| **UHRF1** | GCCATACCCTCTTCGACTACG | GCCCCAATTCCGTCTCATCC |
| **NSD2** | CCCACCATACAAGCACAT | TCAGACACTCCGAATCAAA |
| **KMT5C** | GTTTGACGAGAGCCGAAGGAG | TCGCACAGTTCTCGTGCTGTC |
| **DNMT3A** | ACCGGCCATACGGTGGAG | TATCGTGGTCTTTGGAGGCG |
| **18S** | TTCGTATTGAGCCGCTAGA | CTTTCGCTCTGGTCCGTCTT |
| **GAPDH** | TGACTTCAACAGCGACACCCA | CACCCTGTTGCTGTAGCCAAA |

### Supplementary Table 2. The expression levels of 91 HLM regulators in TCGA-PRAD and normal tissues.

| **Gene** | **Normal (median)** | **Tumor (median)** | **P value** | **P symbol^a^** |
| --- | --- | --- | --- | --- |
| **KMT2E** | 5.125284 | 4.929788 | 0.00056 | *** |
| **SETD1A** | 4.26064 | 4.375905 | 0.272441 | ns |
| **KMT2A** | 3.86801 | 3.75591 | 0.012143 | * |
| **KMT2B** | 4.091941 | 4.390804 | 0.005722 | ** |
| **KMT2C** | 4.097448 | 4.00023 | 0.188716 | ns |
| **KMT2D** | 3.830982 | 4.016728 | 0.356198 | ns |
| **SMYD1** | 0.006717 | 0 | 0.0062 | ** |
| **SMYD2** | 4.198335 | 4.545661 | 1.88E-07 | *** |
| **SETD7** | 5.555048 | 5.406826 | 0.907459 | ns |
| **PRDM9** | 0 | 0 | 0.853229 | ns |
| **SUV39H1** | 3.027019 | 3.053488 | 0.45568 | ns |
| **EHMT2** | 5.042756 | 5.228367 | 0.000324 | *** |
| **EHMT1** | 3.791364 | 3.953574 | 0.010658 | * |
| **SETDB1** | 3.962815 | 4.028307 | 0.217604 | ns |
| **EZH1** | 4.646444 | 4.270219 | 1.38E-07 | *** |
| **EZH2** | 1.494651 | 2.529821 | 4.42E-21 | *** |
| **SETD2** | 5.056522 | 4.950395 | 0.189332 | ns |
| **NSD1** | 4.15853 | 4.056529 | 0.371183 | ns |
| **NSD2** | 2.886121 | 3.200615 | 1.26E-06 | *** |
| **NSD3** | 4.203085 | 3.960136 | 0.000208 | *** |
| **ASH1L** | 4.801442 | 4.536516 | 0.008732 | ** |
| **DOT1L** | 2.501296 | 3.104141 | 5.67E-10 | *** |
| **KMT5A** | 5.411424 | 5.334842 | 0.269651 | ns |
| **KMT5B** | 4.74821 | 4.879254 | 0.099067 | ns |
| **KMT5C** | 2.337107 | 2.941671 | 1.89E-13 | *** |
| **SETDB2** | 3.354046 | 3.161878 | 0.020673 | * |
| **PRDM1** | 2.366466 | 2.216789 | 0.065667 | ns |
| **PRDM13** | 0 | 0 | 0.001534 | ** |
| **PRDM7** | 0.017153 | 0.017998 | 0.511002 | ns |
| **PRDM5** | 2.048018 | 1.321081 | 3.27E-12 | *** |
| **SETD5** | 4.828956 | 4.927078 | 0.420546 | ns |
| **SETD6** | 3.439958 | 3.73626 | 4.02E-05 | *** |
| **PRDM11** | 2.75198 | 1.892982 | 9.33E-18 | *** |
| **PRDM6** | 1.687594 | 1.056907 | 2.54E-07 | *** |
| **PRDM15** | 1.594274 | 1.788588 | 1.04E-05 | *** |
| **SETD1B** | 4.416633 | 4.579955 | 0.133801 | ns |
| **SETMAR** | 4.847824 | 4.862366 | 0.5476 | ns |
| **SETD3** | 5.895265 | 5.837556 | 0.069796 | ns |
| **PRDM10** | 2.800812 | 2.92176 | 0.025345 | * |
| **SMYD4** | 3.296478 | 2.880265 | 1.11E-08 | *** |
| **PRDM2** | 4.352181 | 4.05948 | 0.001676 | ** |
| **SMYD5** | 4.830904 | 4.978257 | 2.18E-05 | *** |
| **SMYD3** | 1.916195 | 2.297704 | 7.66E-12 | *** |
| **SUV39H2** | 3.527289 | 3.604838 | 0.068535 | ns |
| **PRDM16** | 0.609457 | 0.390384 | 3.33E-07 | *** |
| **PRDM8** | 5.258698 | 3.139342 | 3.05E-14 | *** |
| **SETD4** | 2.982827 | 3.010167 | 0.373145 | ns |
| **PRDM12** | 0.058279 | 0.23295 | 2.73E-15 | *** |
| **PRDM4** | 4.556378 | 4.555692 | 0.495297 | ns |
| **MECOM** | 3.454243 | 2.15044 | 8.34E-18 | *** |
| **PRDM14** | 0 | 0 | 0.674821 | ns |
| **KDM1A** | 5.821806 | 6.04958 | 3.65E-06 | *** |
| **KDM1B** | 3.831561 | 3.765868 | 0.956932 | ns |
| **KDM5A** | 4.174654 | 3.926513 | 0.005835 | ** |
| **KDM5B** | 4.47804 | 4.693576 | 0.006415 | ** |
| **KDM5C** | 5.234342 | 5.437221 | 0.018201 | * |
| **KDM5D** | 4.477025 | 4.388418 | 0.656434 | ns |
| **KDM3A** | 3.805061 | 3.726636 | 0.326487 | ns |
| **KDM3B** | 5.636075 | 5.524318 | 0.035914 | * |
| **JMJD1C** | 4.340935 | 4.296026 | 0.395653 | ns |
| **KDM4A** | 5.186202 | 5.213119 | 0.407987 | ns |
| **KDM4B** | 4.266712 | 4.803283 | 1.12E-08 | *** |
| **KDM4C** | 2.594248 | 2.713132 | 0.008732 | ** |
| **KDM4D** | 1.507134 | 1.461972 | 0.699303 | ns |
| **PHF8** | 4.751564 | 5.051094 | 8.84E-06 | *** |
| **PHF2** | 5.583595 | 5.462054 | 0.287928 | ns |
| **KDM6A** | 3.934283 | 4.014207 | 0.411626 | ns |
| **UTY** | 3.590065 | 3.197402 | 0.0001 | *** |
| **KDM6B** | 4.620074 | 4.684809 | 0.736241 | ns |
| **KDM7A** | 3.327438 | 3.630939 | 0.000968 | *** |
| **KDM2A** | 5.213516 | 5.150225 | 0.371183 | ns |
| **KDM2B** | 2.638947 | 2.461279 | 0.007182 | ** |
| **CHD1** | 3.401675 | 3.378833 | 0.333302 | ns |
| **BPTF** | 4.261964 | 4.180518 | 0.56356 | ns |
| **TAF3** | 3.372658 | 2.978768 | 4.45E-13 | *** |
| **SGF29** | 4.641989 | 4.540972 | 0.055983 | ns |
| **ING4** | 5.562653 | 5.572611 | 0.634754 | ns |
| **SPIN1** | 6.360121 | 6.123039 | 4.55E-05 | *** |
| **PHF23** | 5.958076 | 5.974703 | 0.206214 | ns |
| **PYGO2** | 6.082087 | 6.136755 | 0.087472 | ns |
| **UHRF1** | 0.918022 | 1.218638 | 5.8E-06 | *** |
| **CBX7** | 5.732488 | 4.805753 | 5.18E-17 | *** |
| **EED** | 3.046096 | 2.995055 | 0.935802 | ns |
| **BAHD1** | 4.613681 | 4.527653 | 0.02118 | * |
| **DNMT3A** | 2.998192 | 3.255808 | 4.81E-06 | *** |
| **PSIP1** | 5.110251 | 4.56436 | 1.78E-13 | *** |
| **ZMYND11** | 6.468609 | 6.129662 | 6.55E-06 | *** |
| **TP53BP1** | 3.908252 | 3.698031 | 0.096839 | ns |
| **L3MBTL1** | 1.512395 | 1.642238 | 0.081537 | ns |
| **ORC1** | 0.965408 | 1.106213 | 0.002368 | ** |
| **PDP1** | 4.061029 | 4.211566 | 0.010354 | * |

^a^ ns P > 0.05; * P < 0.05; ** P < 0.01; ***P < 0.001.

### Supplementary Table 3. Expression levels of HLM regulators in TCGA-PRAD data stratified by GS.

| **Gene** | **GS < 7 (median)** | **GS = 7 (median)** | **GS > 7 (median)** | **P (GS < 7 vs. GS > 7)** | **P symbol^a^** |
| --- | --- | --- | --- | --- | --- |
| **KMT2E** | 27.40719 | 29.57017 | 29.90849 | 0.020538 | * |
| **SETD1A** | 18.98124 | 19.646115 | 20.04974 | 0.094165 | ns |
| **KMT2A** | 11.78169 | 12.401865 | 12.82618 | 0.139017 | ns |
| **KMT2B** | 18.10854 | 19.103205 | 21.19456 | 0.002183 | ** |
| **KMT2C** | 13.77092 | 14.96071 | 15.30155 | 0.059848 | ns |
| **KMT2D** | 13.76163 | 15.191125 | 15.87215 | 0.06701 | ns |
| **SMYD1** | 0 | 0 | 0 | 0.067015 | ns |
| **SMYD2** | 22.4369 | 21.11062 | 23.18238 | 0.168648 | ns |
| **SETD7** | 39.8987 | 43.154795 | 40.39584 | 0.550631 | ns |
| **PRDM9** | 0 | 0 | 0 | 0.541364 | ns |
| **SUV39H1** | 6.717647 | 7.1762245 | 7.551038 | 0.001719 | ** |
| **EHMT2** | 37.20531 | 35.909755 | 37.74979 | 0.25672 | ns |
| **EHMT1** | 13.84723 | 14.22088 | 15.14204 | 0.010031 | * |
| **SETDB1** | 14.07619 | 14.976145 | 16.23877 | 0.003275 | ** |
| **EZH1** | 18.24918 | 18.07075 | 18.85564 | 0.409099 | ns |
| **EZH2** | 3.682275 | 4.295513 | 5.729042 | 1.57E-09 | *** |
| **SETD2** | 26.95737 | 29.83915 | 31.67322 | 0.019323 | * |
| **NSD1** | 1.26E+01 | 1.47E+01 | 16.52488 | 0.005821 | ** |
| **NSD2** | 6.839394 | 7.5960975 | 9.418541 | 5.71E-07 | *** |
| **NSD3** | 14.01002 | 14.64016 | 14.5087 | 0.073382 | ns |
| **ASH1L** | 2.17E+01 | 2.18E+01 | 22.66214 | 0.27039 | ns |
| **DOT1L** | 6.72E+00 | 7.47E+00 | 8.140263 | 0.006241 | ** |
| **KMT5A** | 42.39655 | 39.520355 | 38.59342 | 0.02848 | * |
| **KMT5B** | 26.60517 | 28.170455 | 29.9395 | 0.015748 | * |
| **KMT5C** | 5.829089 | 6.090603 | 7.693627 | 4.03E-06 | *** |
| **SETDB2** | 8.450087 | 8.01781 | 7.849452 | 0.474164 | ns |
| **PRDM1** | 2.825665 | 3.31207 | 4.341173 | 0.01806 | * |
| **PRDM13** | 0 | 0 | 0 | 0.115029 | ns |
| **PRDM7** | 0.01762386 | 0.012040785 | 0.011145 | 0.00223 | ** |
| **PRDM5** | 1.706809 | 1.4349355 | 1.544867 | 0.173654 | ns |
| **SETD5** | 27.31009 | 28.75975 | 31.76751 | 0.001284 | ** |
| **SETD6** | 13.13207 | 12.27725 | 12.43699 | 0.680635 | ns |
| **PRDM11** | 2.738438 | 2.7175465 | 2.649248 | 0.381146 | ns |
| **PRDM6** | 1.308529 | 1.136754 | 0.94857 | 0.032515 | * |
| **PRDM15** | 2.354387 | 2.433083 | 2.537521 | 0.027341 | * |
| **SETD1B** | 19.76701 | 22.584215 | 24.63904 | 0.001458 | ** |
| **SETMAR** | 29.54828 | 27.74552 | 28.26643 | 0.192442 | ns |
| **SETD3** | 58.55728 | 56.123015 | 55.97204 | 0.8441 | ns |
| **PRDM10** | 5.660666 | 6.5048125 | 7.005276 | 0.003604 | ** |
| **SMYD4** | 6.689671 | 6.4606935 | 6.2628 | 0.959878 | ns |
| **PRDM2** | 16.01937 | 15.64889 | 15.5986 | 0.936211 | ns |
| **SMYD5** | 28.78953 | 30.6689 | 30.94925 | 0.017294 | * |
| **SMYD3** | 3.621219 | 3.906413 | 3.996133 | 0.115141 | ns |
| **SUV39H2** | 12.64365 | 11.680895 | 10.27801 | 0.033837 | * |
| **PRDM16** | 0.2867863 | 0.2880547 | 0.356468 | 0.138407 | ns |
| **PRDM8** | 9.369426 | 9.224155 | 6.66341 | 0.021819 | * |
| **SETD4** | 6.829223 | 6.675925 | 7.497789 | 0.011363 | * |
| **PRDM12** | 0.1259596 | 0.1410286 | 0.221266 | 2.94E-05 | *** |
| **PRDM4** | 21.17998 | 22.62852 | 22.36337 | 0.18857 | ns |
| **MECOM** | 2.464508 | 3.4794165 | 3.79071 | 0.001017 | ** |
| **PRDM14** | 0 | 0 | 0 | 0.81895 | ns |
| **KDM1A** | 59.94381 | 65.553515 | 66.31334 | 0.204409 | ns |
| **KDM1B** | 11.28393 | 12.96148 | 12.59087 | 0.090623 | ns |
| **KDM5A** | 13.64152 | 14.1728 | 14.29859 | 0.183999 | ns |
| **KDM5B** | 20.60548 | 24.662325 | 25.86454 | 0.001705 | ** |
| **KDM5C** | 38.56553 | 41.42848 | 43.99186 | 0.002561 | ** |
| **KDM5D** | 20.09912 | 19.134305 | 20.12066 | 0.550631 | ns |
| **KDM3A** | 11.40755 | 11.966245 | 12.80953 | 0.058009 | ns |
| **KDM3B** | 40.16385 | 44.1164 | 45.25071 | 0.079847 | ns |
| **JMJD1C** | 16.20386 | 18.665655 | 18.81281 | 0.022893 | * |
| **KDM4A** | 36.09413 | 36.810595 | 35.76413 | 0.66894 | ns |
| **KDM4B** | 27.0228 | 27.843455 | 25.76974 | 0.59735 | ns |
| **KDM4C** | 5.572259 | 5.3994655 | 5.804462 | 0.068733 | ns |
| **KDM4D** | 1.645255 | 1.7521635 | 1.836571 | 0.085097 | ns |
| **PHF8** | 27.52965 | 34.31722 | 31.14366 | 0.499955 | ns |
| **PHF2** | 38.08987 | 42.627625 | 45.4758 | 0.015261 | * |
| **KDM6A** | 12.86504 | 15.15283 | 15.76406 | 0.015552 | * |
| **UTY** | 7.387336 | 8.3314355 | 8.1982 | 0.069784 | ns |
| **KDM6B** | 21.58861 | 24.745305 | 25.05485 | 0.095521 | ns |
| **KDM7A** | 8.882304 | 11.4795 | 11.54341 | 0.005128 | ** |
| **KDM2A** | 30.83865 | 33.27872 | 36.59872 | 0.000574 | *** |
| **KDM2B** | 3.875457 | 4.258702 | 4.85279 | 0.000769 | *** |
| **CHD1** | 8.798913 | 9.113387 | 9.885812 | 0.205226 | ns |
| **BPTF** | 15.23886 | 17.09717 | 17.66482 | 0.055048 | ns |
| **TAF3** | 6.749955 | 6.8801335 | 6.92575 | 0.488401 | ns |
| **SGF29** | 23.60111 | 22.51228 | 22.07185 | 0.308888 | ns |
| **ING4** | 46.02409 | 46.39932 | 47.23546 | 0.391173 | ns |
| **SPIN1** | 60.9242 | 66.27617 | 72.35832 | 0.001627 | ** |
| **PHF23** | 61.89693 | 65.476705 | 58.88087 | 0.290771 | ns |
| **PYGO2** | 63.85155 | 68.981625 | 70.61353 | 0.001976 | ** |
| **UHRF1** | 1.036507 | 1.1738145 | 1.856812 | 1.13E-07 | *** |
| **CBX7** | 30.29865 | 28.037555 | 25.57461 | 0.156941 | ns |
| **EED** | 6.288189 | 6.765655 | 7.475823 | 3.26E-05 | *** |
| **BAHD1** | 21.06067 | 21.945645 | 22.47577 | 0.134197 | ns |
| **DNMT3A** | 7.097349 | 8.079932 | 9.897932 | 0.000331 | *** |
| **PSIP1** | 22.17912 | 22.200375 | 23.2248 | 0.371279 | ns |
| **ZMYND11** | 64.485395 | 69.561685 | 68.84439 | 0.572208 | ns |
| **TP53BP1** | 10.52953 | 11.94334 | 12.22748 | 0.057107 | ns |
| **L3MBTL1** | 1.944062 | 1.791366 | 2.484536 | 0.002392 | ** |
| **ORC1** | 0.9271376 | 1.068369 | 1.438582 | 7.52E-08 | *** |
| **PDP1** | 14.94699 | 17.53331 | 18.10513 | 0.023729 | * |

^a^ ns P > 0.05; * P < 0.05; ** P < 0.01; ***P < 0.001.

### Supplementary Table 4. Expression levels of HLM regulators in TCGA-PRAD data stratified by pT stage.

| **Gene** | **pT = 2 (median)** | **pT = 3 (median)** | **pT = 4 (median)** | **P (pT = 2 vs. pT = 3)** | **P symbol^a^** |
| --- | --- | --- | --- | --- | --- |
| **KMT2E** | 28.48013 | 29.74464 | 30.87119 | 0.162093 | ns |
| **SETD1A** | 19.49918 | 19.78676 | 24.32138 | 0.35348 | ns |
| **KMT2A** | 12.16831 | 12.52165 | 14.11666 | 0.51722 | ns |
| **KMT2B** | 19.53902 | 20.00268 | 24.99531 | 0.080668 | ns |
| **KMT2C** | 14.91491 | 15.06734 | 17.95644 | 0.756759 | ns |
| **KMT2D** | 14.85816 | 15.14782 | 19.93158 | 0.406451 | ns |
| **SMYD1** | 0 | 0 | 0 | 0.491408 | ns |
| **SMYD2** | 21.0706 | 22.7853 | 24.96923 | 0.033269 | * |
| **SETD7** | 42.51433 | 40.75677 | 43.49664 | 0.545252 | ns |
| **PRDM9** | 0 | 0 | 0 | 0.92188 | ns |
| **SUV39H1** | 7.010256 | 7.456561 | 8.403733 | 0.004542 | ** |
| **EHMT2** | 35.45154 | 37.24469 | 38.81833 | 0.157259 | ns |
| **EHMT1** | 14.12782 | 14.79485 | 17.05668 | 0.056002 | ns |
| **SETDB1** | 14.54912 | 15.76442 | 21.2089 | 0.006205 | ** |
| **EZH1** | 18.16295 | 18.39898 | 18.18165 | 0.804138 | ns |
| **EZH2** | 4.21339 | 5.141916 | 6.159131 | 2.28E-07 | *** |
| **SETD2** | 29.09451 | 30.79412 | 37.18086 | 0.122273 | ns |
| **NSD1** | 1.42E+01 | 16.35866 | 22.85756 | 0.021925 | * |
| **NSD2** | 7.440956 | 8.949861 | 12.07481 | 3.01E-06 | *** |
| **NSD3** | 14.49716 | 14.53911 | 17.58849 | 0.645252 | ns |
| **ASH1L** | 2.22E+01 | 21.8572 | 34.09696 | 0.527367 | ns |
| **DOT1L** | 7.41E+00 | 7.83959 | 9.162032 | 0.110446 | ns |
| **KMT5A** | 41.19217 | 38.68931 | 36.12801 | 0.003601 | ** |
| **KMT5B** | 27.30177 | 29.18719 | 34.6118 | 0.086982 | ns |
| **KMT5C** | 6.11489 | 6.942208 | 7.582606 | 0.000381 | *** |
| **SETDB2** | 7.800756 | 8.046505 | 9.537885 | 0.643305 | ns |
| **PRDM1** | 3.146559 | 3.995227 | 4.526905 | 0.034178 | * |
| **PRDM13** | 0 | 0 | 0 | 0.354998 | ns |
| **PRDM7** | 0.01231615 | 0.012483 | 0.012486 | 0.699112 | ns |
| **PRDM5** | 1.4631 | 1.504921 | 1.464717 | 0.550675 | ns |
| **SETD5** | 27.75218 | 30.4634 | 39.11577 | 0.013228 | * |
| **SETD6** | 12.67221 | 12.11101 | 13.4431 | 0.905751 | ns |
| **PRDM11** | 2.714022 | 2.647323 | 3.139358 | 0.37281 | ns |
| **PRDM6** | 1.109603 | 1.04141 | 0.82404 | 0.52471 | ns |
| **PRDM15** | 2.379561 | 2.481471 | 2.744102 | 0.121618 | ns |
| **SETD1B** | 22.00579 | 23.82688 | 28.80473 | 0.038408 | * |
| **SETMAR** | 27.59367 | 28.3881 | 33.511 | 0.52648 | ns |
| **SETD3** | 57.80459 | 55.40538 | 58.14914 | 0.414942 | ns |
| **PRDM10** | 6.399005 | 6.70783 | 8.478378 | 0.053942 | ns |
| **SMYD4** | 6.647712 | 6.198572 | 7.687165 | 0.163729 | ns |
| **PRDM2** | 16.00658 | 15.47227 | 16.8726 | 0.597818 | ns |
| **SMYD5** | 30.41042 | 30.66774 | 29.00143 | 0.250813 | ns |
| **SMYD3** | 3.67382 | 4.024607 | 5.204009 | 0.03612 | * |
| **SUV39H2** | 11.9978 | 10.64469 | 10.37125 | 0.01567 | * |
| **PRDM16** | 0.2758981 | 0.33485 | 0.441802 | 0.026207 | * |
| **PRDM8** | 9.297093 | 7.002374 | 3.710963 | 0.004134 | ** |
| **SETD4** | 6.92341 | 7.194307 | 6.378336 | 0.061159 | ns |
| **PRDM12** | 0.1337199 | 0.196654 | 0.261765 | 0.000865 | *** |
| **PRDM4** | 22.50517 | 22.44447 | 26.06004 | 0.451975 | ns |
| **MECOM** | 3.036639 | 3.887526 | 4.582646 | 0.007308 | ** |
| **PRDM14** | 0 | 0 | 0 | 0.736417 | ns |
| **KDM1A** | 63.6827 | 66.44071 | 78.53557 | 0.409141 | ns |
| **KDM1B** | 12.104 | 12.74604 | 17.43597 | 0.282027 | ns |
| **KDM5A** | 14.3488 | 13.92175 | 17.96368 | 0.815182 | ns |
| **KDM5B** | 23.13123 | 25.48477 | 36.20191 | 0.017192 | * |
| **KDM5C** | 41.40797 | 42.94368 | 54.51642 | 0.067208 | ns |
| **KDM5D** | 20.37321 | 19.61657 | 17.80501 | 0.439427 | ns |
| **KDM3A** | 11.80804 | 12.28788 | 14.40003 | 0.159461 | ns |
| **KDM3B** | 43.1044 | 45.03908 | 54.90221 | 0.426293 | ns |
| **JMJD1C** | 18.11472 | 18.81213 | 21.52196 | 0.126934 | ns |
| **KDM4A** | 36.4673 | 35.8707 | 40.57893 | 0.483577 | ns |
| **KDM4B** | 27.66455 | 26.50245 | 27.28701 | 0.197694 | ns |
| **KDM4C** | 5.37045 | 5.623503 | 6.417405 | 0.116952 | ns |
| **KDM4D** | 1.657344 | 1.823594 | 1.925983 | 0.272132 | ns |
| **PHF8** | 32.82317 | 30.8882 | 43.65279 | 0.323363 | ns |
| **PHF2** | 41.88532 | 44.82234 | 48.58286 | 0.034582 | * |
| **KDM6A** | 14.37953 | 15.79606 | 19.63228 | 0.044494 | * |
| **UTY** | 8.365039 | 7.950071 | 8.72313 | 0.981866 | ns |
| **KDM6B** | 24.65569 | 24.71982 | 23.38445 | 0.85278 | ns |
| **KDM7A** | 10.83419 | 11.48746 | 15.3404 | 0.072438 | ns |
| **KDM2A** | 32.33783 | 35.56318 | 41.34292 | 0.009611 | ** |
| **KDM2B** | 4.090784 | 4.730426 | 6.113811 | 0.002525 | ** |
| **CHD1** | 9.408752 | 9.346997 | 11.92635 | 0.798893 | ns |
| **BPTF** | 16.39929 | 17.21195 | 25.82451 | 0.460176 | ns |
| **TAF3** | 6.883125 | 6.856599 | 7.411739 | 0.549317 | ns |
| **SGF29** | 22.09796 | 22.27924 | 21.79407 | 0.847462 | ns |
| **ING4** | 46.59082 | 46.43811 | 50.99544 | 0.643792 | ns |
| **SPIN1** | 64.79062 | 71.41831 | 86.73411 | 0.002736 | ** |
| **PHF23** | 65.09288 | 60.65281 | 49.23939 | 0.01567 | * |
| **PYGO2** | 67.48181 | 69.8393 | 90.48282 | 0.007412 | ** |
| **UHRF1** | 1.059026 | 1.68256 | 2.168395 | 2.66E-09 | *** |
| **CBX7** | 27.69158 | 26.76726 | 28.25235 | 0.239246 | ns |
| **EED** | 6.714755 | 7.156008 | 8.938476 | 5.11E-05 | *** |
| **BAHD1** | 22.2552 | 21.54237 | 25.5148 | 0.218265 | ns |
| **DNMT3A** | 7.697076 | 9.238869 | 10.47764 | 5.53E-05 | *** |
| **PSIP1** | 22.26648 | 22.61437 | 29.49475 | 0.187049 | ns |
| **ZMYND11** | 69.47556 | 69.01837 | 68.1371 | 0.958063 | ns |
| **TP53BP1** | 11.8954 | 11.9821 | 11.66067 | 0.929443 | ns |
| **L3MBTL1** | 1.970446 | 2.21481 | 3.373673 | 0.034991 | * |
| **ORC1** | 1.031816 | 1.327388 | 2.183189 | 4.28E-07 | *** |
| **PDP1** | 16.03609 | 18.11951 | 21.86397 | 0.087232 | ns |

^a^ ns P > 0.05; * P < 0.05; ** P < 0.01; ***P < 0.001.

### Supplementary Table 5. Expression levels of HLM regulators in TCGA-PRAD data stratified by TP53 mutation.

| **Gene** | **Wild type (median)** | **Mutation (median)** | **P value (wild vs. mut)** | **P symbol^a^** |
| --- | --- | --- | --- | --- |
| **KMT2E** | 28.74054 | 31.66381 | 0.023215 | * |
| **SETD1A** | 19.50251 | 22.03162 | 0.008336 | ** |
| **KMT2A** | 12.0905 | 14.14522 | 0.023498 | * |
| **KMT2B** | 19.3696 | 21.82006 | 0.029811 | * |
| **KMT2C** | 14.70966 | 16.60477 | 0.073572 | ns |
| **KMT2D** | 14.70546 | 17.67332 | 0.056171 | ns |
| **SMYD1** | 0 | 0 | 0.863081 | ns |
| **SMYD2** | 21.97321 | 28.43999 | 0.000918 | *** |
| **SETD7** | 42.02723 | 37.76863 | 0.758922 | ns |
| **PRDM9** | 0 | 0 | 0.425313 | ns |
| **SUV39H1** | 7.271543 | 7.630504 | 0.242129 | ns |
| **EHMT2** | 36.16907 | 38.6237 | 0.046633 | * |
| **EHMT1** | 14.35298 | 15.59029 | 0.015453 | * |
| **SETDB1** | 15.08687 | 17.22324 | 0.011658 | * |
| **EZH1** | 18.04829 | 18.72867 | 0.741356 | ns |
| **EZH2** | 4.717047 | 6.624073 | 1.28E-05 | *** |
| **SETD2** | 29.09451 | 35.85469 | 0.003339 | ** |
| **NSD1** | 15.05776 | 19.72604 | 0.017155 | * |
| **NSD2** | 8.193508 | 9.433184 | 0.013546 | * |
| **NSD3** | 14.33428 | 15.46249 | 0.15735 | ns |
| **ASH1L** | 21.23278 | 25.53618 | 0.083594 | ns |
| **DOT1L** | 7.561815 | 8.680385 | 0.124059 | ns |
| **KMT5A** | 39.00566 | 40.25657 | 0.982445 | ns |
| **KMT5B** | 28.13773 | 34.03223 | 0.003402 | ** |
| **KMT5C** | 6.572497 | 7.651272 | 0.012987 | * |
| **SETDB2** | 7.71639 | 8.297667 | 0.308142 | ns |
| **PRDM1** | 3.47007 | 3.722749 | 0.299969 | ns |
| **PRDM13** | 0 | 0 | 0.256761 | ns |
| **PRDM7** | 0.01227734 | 0.014179 | 0.253281 | ns |
| **PRDM5** | 1.438163 | 1.376751 | 0.948291 | ns |
| **SETD5** | 28.88383 | 37.18439 | 0.001258 | ** |
| **SETD6** | 12.29114 | 12.79786 | 0.773066 | ns |
| **PRDM11** | 2.647323 | 2.641639 | 0.812338 | ns |
| **PRDM6** | 1.02968 | 0.945091 | 0.931705 | ns |
| **PRDM15** | 2.425282 | 2.776149 | 0.033192 | * |
| **SETD1B** | 22.58329 | 26.58583 | 0.012448 | * |
| **SETMAR** | 28.17719 | 28.74269 | 0.698043 | ns |
| **SETD3** | 56.4653 | 56.43404 | 0.490779 | ns |
| **PRDM10** | 6.571228 | 7.989573 | 0.005142 | ** |
| **SMYD4** | 6.362856 | 5.141913 | 0.020358 | * |
| **PRDM2** | 15.27088 | 16.46721 | 0.243999 | ns |
| **SMYD5** | 30.67006 | 31.53443 | 0.482076 | ns |
| **SMYD3** | 3.962243 | 4.251752 | 0.373155 | ns |
| **SUV39H2** | 11.21058 | 11.34624 | 0.705772 | ns |
| **PRDM16** | 0.2943451 | 0.358258 | 0.046506 | * |
| **PRDM8** | 7.50264 | 6.273697 | 0.172491 | ns |
| **SETD4** | 7.027984 | 6.980053 | 0.534007 | ns |
| **PRDM12** | 0.1803294 | 0.269977 | 0.004802 | ** |
| **PRDM4** | 22.31332 | 22.20517 | 0.637393 | ns |
| **MECOM** | 3.194339 | 4.444881 | 0.015063 | * |
| **PRDM14** | 0 | 0 | 0.696116 | ns |
| **KDM1A** | 65.0401 | 77.15068 | 0.007624 | ** |
| **KDM1B** | 12.36455 | 14.84567 | 0.015902 | * |
| **KDM5A** | 14.04685 | 14.42468 | 0.514394 | ns |
| **KDM5B** | 24.72946 | 32.25909 | 0.004323 | ** |
| **KDM5C** | 41.62159 | 47.65436 | 0.006385 | ** |
| **KDM5D** | 19.61657 | 18.7751 | 0.908721 | ns |
| **KDM3A** | 12.07111 | 13.91303 | 0.016105 | * |
| **KDM3B** | 43.58418 | 47.90914 | 0.141976 | ns |
| **JMJD1C** | 18.51373 | 20.333 | 0.018726 | * |
| **KDM4A** | 35.88997 | 38.5516 | 0.256886 | ns |
| **KDM4B** | 27.11506 | 28.15716 | 0.968591 | ns |
| **KDM4C** | 5.488577 | 5.833056 | 0.453682 | ns |
| **KDM4D** | 1.718173 | 2.078176 | 0.008422 | ** |
| **PHF8** | 32.77429 | 31.0508 | 0.901381 | ns |
| **PHF2** | 42.60361 | 57.37598 | 0.001881 | ** |
| **KDM6A** | 14.81995 | 19.29884 | 0.003733 | ** |
| **UTY** | 8.100264 | 7.766226 | 0.866643 | ns |
| **KDM6B** | 24.40947 | 25.19411 | 0.704911 | ns |
| **KDM7A** | 11.32656 | 14.10595 | 0.003052 | ** |
| **KDM2A** | 33.2163 | 40.36291 | 0.00142 | ** |
| **KDM2B** | 4.392345 | 4.77674 | 0.041989 | * |
| **CHD1** | 9.241339 | 10.29641 | 0.118222 | ns |
| **BPTF** | 16.75536 | 19.38354 | 0.057679 | ns |
| **TAF3** | 6.812832 | 7.010707 | 0.381919 | ns |
| **SGF29** | 22.34265 | 21.19206 | 0.034161 | * |
| **ING4** | 46.8143 | 44.03601 | 0.339359 | ns |
| **SPIN1** | 68.28573 | 73.88238 | 0.021912 | * |
| **PHF23** | 62.12689 | 48.09831 | 1.35E-05 | *** |
| **PYGO2** | 69.32243 | 70.29102 | 0.383813 | ns |
| **UHRF1** | 1.327117 | 2.644231 | 0.000739 | *** |
| **CBX7** | 26.78431 | 24.16974 | 0.079143 | ns |
| **EED** | 6.868956 | 8.28326 | 0.002515 | ** |
| **BAHD1** | 21.61226 | 23.64342 | 0.086744 | ns |
| **DNMT3A** | 8.44966 | 11.51382 | 0.000907 | *** |
| **PSIP1** | 22.17912 | 22.99567 | 0.543954 | ns |
| **ZMYND11** | 67.91427 | 75.32712 | 0.018038 | * |
| **TP53BP1** | 11.85831 | 13.68949 | 0.026101 | * |
| **L3MBTL1** | 2.053927 | 2.628183 | 0.021979 | * |
| **ORC1** | 1.137293 | 1.578951 | 0.002574 | ** |
| **PDP1** | 17.21819 | 24.70662 | 0.002198 | ** |

^a^ ns P > 0.05; * P < 0.05; ** P < 0.01; ***P < 0.001.

### Supplementary Table 6. Abundance of each infiltrating immune cell among the three HLMclusters.

| **Immune cell infiltration** | **Group A (median)** | **Group B (median)** | **Group C (median)** | **P value** | **P symbol^a^** |
| --- | --- | --- | --- | --- | --- |
| **Activated.B.cellna** | 0.292357 | 0.316045 | 0.29528 | 0.327794 | ns |
| **Activated.CD4.T.cellna** | 0.518148 | 0.473598 | 0.484761 | 3.81E-06 | *** |
| **Activated.CD8.T.cellna** | 0.670714 | 0.707091 | 0.672326 | 0.000438 | *** |
| **Activated.dendritic.cellna** | 0.549066 | 0.542414 | 0.536563 | 0.048805 | * |
| **CD56bright.natural.killer.cellna** | 0.766733 | 0.771846 | 0.771997 | 0.017727 | * |
| **CD56dim.natural.killer.cellna** | 0.71876 | 0.738914 | 0.732865 | 1.26E-09 | *** |
| **Eosinophilna** | 0.395822 | 0.382646 | 0.376006 | 0.000391 | *** |
| **Gamma.delta.T.cellna** | 0.630377 | 0.623989 | 0.629023 | 0.67399 | ns |
| **Immature..B.cellna** | 0.383118 | 0.358273 | 0.367126 | 0.081739 | ns |
| **Immature.dendritic.cellna** | 0.77843 | 0.758493 | 0.769071 | 1.09E-06 | *** |
| **MDSCna** | 0.669571 | 0.686638 | 0.657927 | 0.056659 | ns |
| **Macrophagena** | 0.427826 | 0.444456 | 0.428018 | 0.005929 | ** |
| **Mast.cellna** | 0.495092 | 0.499867 | 0.490924 | 0.605259 | ns |
| **Monocytena** | 0.868917 | 0.870788 | 0.865985 | 0.011036 | * |
| **Natural.killer.T.cellna** | 0.419809 | 0.430267 | 0.422129 | 0.211941 | ns |
| **Natural.killer.cellna** | 0.712274 | 0.698501 | 0.7041 | 0.040181 | * |
| **Neutrophilna** | 0.200037 | 0.200269 | 0.19127 | 0.075848 | ns |
| **Plasmacytoid.dendritic.cellna** | 0.773056 | 0.766382 | 0.772332 | 0.948972 | ns |
| **Regulatory.T.cellna** | 0.523058 | 0.486861 | 0.490795 | 3.77E-05 | *** |
| **T.follicular.helper.cellna** | 0.555454 | 0.551429 | 0.552154 | 0.85854 | ns |
| **Type.1.T.helper.cellna** | 0.533994 | 0.531324 | 0.531043 | 0.945414 | ns |
| **Type.17.T.helper.cellna** | 0.399224 | 0.420393 | 0.406865 | 0.000829 | *** |
| **Type.2.T.helper.cellna** | 0.544272 | 0.501969 | 0.532755 | 1.28E-14 | *** |

^a^ ns P > 0.05; * P < 0.05; ** P < 0.01; ***P < 0.001.

### Supplementary Table 7. Abundance of each infiltrating immune cell among the three geneClusters.

| **Immune cell infiltration** | **Group A (median)** | **Group B (median)** | **Group C (median)** | **P value** | **P symbol^a^** |
| --- | --- | --- | --- | --- | --- |
| **Activated.B.cellna** | 0.293491 | 0.304176 | 0.30282 | 0.928275 | ns |
| **Activated.CD4.T.cellna** | 0.527565 | 0.482457 | 0.470025 | 1.6E-10 | *** |
| **Activated.CD8.T.cellna** | 0.67401 | 0.677109 | 0.682604 | 0.584107 | ns |
| **Activated.dendritic.cellna** | 0.550477 | 0.541611 | 0.530508 | 0.058984 | ns |
| **CD56bright.natural.killer.cellna** | 0.768474 | 0.771846 | 0.76357 | 0.080386 | ns |
| **CD56dim.natural.killer.cellna** | 0.73092 | 0.724631 | 0.739293 | 0.005066 | ** |
| **Eosinophilna** | 0.391757 | 0.381289 | 0.360063 | 0.001071 | ** |
| **Gamma.delta.T.cellna** | 0.628806 | 0.630192 | 0.625132 | 0.453914 | ns |
| **Immature..B.cellna** | 0.390418 | 0.367134 | 0.350114 | 0.100225 | ns |
| **Immature.dendritic.cellna** | 0.772006 | 0.77415 | 0.750524 | 2.53E-07 | *** |
| **MDSCna** | 0.678215 | 0.658118 | 0.660829 | 0.564127 | ns |
| **Macrophagena** | 0.432002 | 0.429837 | 0.437769 | 0.595872 | ns |
| **Mast.cellna** | 0.491496 | 0.501212 | 0.484071 | 0.226761 | ns |
| **Monocytena** | 0.867082 | 0.869691 | 0.866475 | 0.060645 | ns |
| **Natural.killer.T.cellna** | 0.420952 | 0.424328 | 0.419346 | 0.380244 | ns |
| **Natural.killer.cellna** | 0.708621 | 0.706974 | 0.692305 | 0.056285 | ns |
| **Neutrophilna** | 0.188901 | 0.198823 | 0.205671 | 0.10164 | ns |
| **Plasmacytoid.dendritic.cellna** | 0.770769 | 0.774069 | 0.766382 | 0.144834 | ns |
| **Regulatory.T.cellna** | 0.527253 | 0.491207 | 0.478951 | 2.29E-07 | *** |
| **T.follicular.helper.cellna** | 0.554519 | 0.554584 | 0.542317 | 0.188284 | ns |
| **Type.1.T.helper.cellna** | 0.535424 | 0.532533 | 0.520345 | 0.261163 | ns |
| **Type.17.T.helper.cellna** | 0.393822 | 0.41571 | 0.41312 | 3.59E-08 | *** |
| **Type.2.T.helper.cellna** | 0.548343 | 0.524773 | 0.502639 | 1.44E-11 | *** |

^a^ ns P > 0.05; ** P < 0.01; ***P < 0.001.

### Supplementary Table 8. Expression of HLM regulators among the three geneClusters.

| **Gene** | **Group A (median)** | **Group B (median)** | **Group C (median)** | **P value** | **P symbol^a^** |
| --- | --- | --- | --- | --- | --- |
| **KMT2E** | 34.87137 | 26.41965 | 17.97675 | 4.9E-28 | *** |
| **SETD1A** | 22.88316 | 18.00526 | 15.58188 | 7.43E-35 | *** |
| **KMT2A** | 15.91546 | 10.94124 | 6.999935 | 1.94E-34 | *** |
| **KMT2B** | 24.58113 | 17.54569 | 12.99276 | 2.27E-37 | *** |
| **KMT2C** | 18.49435 | 13.38639 | 8.86212 | 6.87E-28 | *** |
| **KMT2D** | 21.63342 | 12.59264 | 7.705073 | 2.44E-36 | *** |
| **SMYD1** | 0 | 0 | 0 | 0.021955 | * |
| **SMYD2** | 25.20464 | 20.48151 | 19.01243 | 6.52E-08 | *** |
| **SETD7** | 50.76265 | 38.13891 | 28.69764 | 3.05E-18 | *** |
| **PRDM9** | 0 | 0 | 0 | 0.076326 | ns |
| **SUV39H1** | 7.543185 | 7.015159 | 7.322435 | 0.000561 | *** |
| **EHMT2** | 41.26534 | 33.38046 | 33.50633 | 1.54E-19 | *** |
| **EHMT1** | 17.22356 | 12.88993 | 10.60042 | 8.58E-34 | *** |
| **SETDB1** | 19.39648 | 13.39546 | 10.09684 | 9.45E-53 | *** |
| **EZH1** | 20.04817 | 17.86605 | 16.36605 | 1.83E-06 | *** |
| **EZH2** | 5.79709 | 4.089599 | 3.817788 | 1.02E-19 | *** |
| **SETD2** | 38.93454 | 26.60392 | 19.27181 | 1.58E-41 | *** |
| **NSD1** | 21.32103 | 12.83556 | 8.389878 | 8.3E-46 | *** |
| **NSD2** | 10.50317 | 7.080037 | 5.411837 | 5.81E-51 | *** |
| **NSD3** | 17.75225 | 13.64332 | 9.88148 | 2.45E-19 | *** |
| **ASH1L** | 29.97718 | 19.28364 | 10.82318 | 2.3E-37 | *** |
| **DOT1L** | 10.13351 | 6.755406 | 5.305119 | 5.71E-33 | *** |
| **KMT5A** | 40.58791 | 38.83254 | 37.45696 | 0.008695 | ** |
| **KMT5B** | 33.59647 | 25.85601 | 20.48362 | 5.17E-37 | *** |
| **KMT5C** | 7.332904 | 6.277166 | 6.185009 | 5.93E-07 | *** |
| **SETDB2** | 9.157753 | 7.257656 | 5.743655 | 7.53E-19 | *** |
| **PRDM1** | 4.809589 | 3.354444 | 1.834655 | 7.53E-12 | *** |
| **PRDM13** | 0 | 0 | 0 | 0.000248 | *** |
| **PRDM7** | 0.013483 | 0.010812 | 0.012483 | 0.434963 | ns |
| **PRDM5** | 1.733361 | 1.498533 | 0.897893 | 2.51E-09 | *** |
| **SETD5** | 38.01029 | 26.41216 | 17.71847 | 1.33E-42 | *** |
| **SETD6** | 14.2176 | 11.37076 | 9.442869 | 2.08E-16 | *** |
| **PRDM11** | 3.318677 | 2.557272 | 1.800597 | 2.96E-16 | *** |
| **PRDM6** | 1.08531 | 1.165261 | 0.915217 | 0.037672 | * |
| **PRDM15** | 3.00716 | 2.252396 | 1.955329 | 2.37E-30 | *** |
| **SETD1B** | 28.68172 | 19.61829 | 15.14441 | 1.16E-38 | *** |
| **SETMAR** | 30.73713 | 26.71042 | 25.74405 | 3.24E-06 | *** |
| **SETD3** | 60.49019 | 54.2938 | 50.27785 | 2.35E-09 | *** |
| **PRDM10** | 8.450753 | 5.581594 | 3.790477 | 2.54E-40 | *** |
| **SMYD4** | 7.606222 | 6.000315 | 4.709033 | 7.58E-18 | *** |
| **PRDM2** | 18.88796 | 14.40113 | 9.69983 | 1.96E-26 | *** |
| **SMYD5** | 32.18204 | 29.33286 | 29.65562 | 1.95E-05 | *** |
| **SMYD3** | 4.630562 | 3.583263 | 3.373673 | 2.43E-13 | *** |
| **SUV39H2** | 11.98001 | 10.74403 | 10.47509 | 0.127201 | ns |
| **PRDM16** | 0.35616 | 0.302465 | 0.252453 | 0.00083 | *** |
| **PRDM8** | 7.307835 | 9.074146 | 6.603909 | 0.001171 | ** |
| **SETD4** | 7.730101 | 6.675777 | 6.15241 | 2.8E-11 | *** |
| **PRDM12** | 0.203149 | 0.149557 | 0.123914 | 4.55E-05 | *** |
| **PRDM4** | 24.90709 | 21.67293 | 17.81778 | 1.15E-13 | *** |
| **MECOM** | 4.323171 | 3.261318 | 1.880718 | 3.14E-12 | *** |
| **PRDM14** | 0 | 0 | 0 | 0.084638 | ns |
| **KDM1A** | 79.85926 | 60.02412 | 52.56209 | 1.26E-33 | *** |
| **KDM1B** | 15.60041 | 11.37534 | 8.199799 | 7.43E-24 | *** |
| **KDM5A** | 17.87168 | 12.65015 | 8.682393 | 2.27E-29 | *** |
| **KDM5B** | 35.11885 | 21.7083 | 16.00492 | 1.23E-47 | *** |
| **KDM5C** | 51.56615 | 37.88827 | 27.24733 | 1.11E-44 | *** |
| **KDM5D** | 25.2006 | 17.78557 | 13.26558 | 2.28E-33 | *** |
| **KDM3A** | 15.5227 | 10.96656 | 8.1654 | 7.31E-37 | *** |
| **KDM3B** | 54.86053 | 39.64572 | 28.02839 | 1.2E-34 | *** |
| **JMJD1C** | 25.48573 | 16.01201 | 8.347572 | 2.22E-39 | *** |
| **KDM4A** | 44.91822 | 31.99041 | 26.28088 | 1.45E-33 | *** |
| **KDM4B** | 30.78348 | 25.91664 | 21.85916 | 1.2E-13 | *** |
| **KDM4C** | 6.281112 | 5.179228 | 4.854586 | 6.59E-21 | *** |
| **KDM4D** | 2.123607 | 1.534619 | 1.216991 | 8.41E-22 | *** |
| **PHF8** | 37.35212 | 29.72217 | 25.73411 | 4.21E-08 | *** |
| **PHF2** | 58.90617 | 38.45422 | 24.34172 | 1.51E-54 | *** |
| **KDM6A** | 21.7154 | 12.81758 | 7.841096 | 2.53E-39 | *** |
| **UTY** | 10.17101 | 7.305711 | 5.287992 | 4.36E-31 | *** |
| **KDM6B** | 30.3312 | 22.31103 | 16.63685 | 4.99E-24 | *** |
| **KDM7A** | 14.83107 | 9.796422 | 6.484587 | 3.14E-31 | *** |
| **KDM2A** | 41.88665 | 30.84841 | 22.63615 | 3.77E-41 | *** |
| **KDM2B** | 5.48977 | 4.017202 | 2.934871 | 6.28E-38 | *** |
| **CHD1** | 12.6603 | 8.317571 | 4.935027 | 1.18E-32 | *** |
| **BPTF** | 23.15893 | 14.86 | 9.531079 | 7.48E-41 | *** |
| **TAF3** | 7.653053 | 6.688176 | 5.509233 | 8.14E-15 | *** |
| **SGF29** | 22.03534 | 22.09796 | 24.77286 | 0.011318 | * |
| **ING4** | 49.36793 | 45.02427 | 47.42613 | 0.000633 | *** |
| **SPIN1** | 83.97577 | 63.06478 | 43.18676 | 1.43E-42 | *** |
| **PHF23** | 59.69094 | 65.03082 | 60.81969 | 0.068359 | ns |
| **PYGO2** | 75.5308 | 66.29514 | 57.70549 | 2.49E-20 | *** |
| **UHRF1** | 2.238536 | 1.12376 | 0.724973 | 2.06E-25 | *** |
| **CBX7** | 25.83679 | 29.59193 | 24.42065 | 0.020824 | * |
| **EED** | 8.004559 | 6.662327 | 6.088265 | 5.69E-22 | *** |
| **BAHD1** | 24.09864 | 21.02267 | 17.92553 | 6.73E-11 | *** |
| **DNMT3A** | 12.2799 | 7.525324 | 5.531315 | 3.13E-51 | *** |
| **PSIP1** | 24.90333 | 22.13268 | 16.54481 | 2.73E-13 | *** |
| **ZMYND11** | 80.9602 | 64.06763 | 46.01942 | 2.26E-28 | *** |
| **TP53BP1** | 16.54244 | 10.47981 | 6.235529 | 2.7E-37 | *** |
| **L3MBTL1** | 2.593468 | 1.914527 | 1.659663 | 5.66E-12 | *** |
| **ORC1** | 1.604374 | 0.959656 | 0.894911 | 1.19E-26 | *** |
| **PDP1** | 25.29922 | 15.11439 | 9.305824 | 1.46E-39 | *** |

^a^ ns P > 0.05; * P < 0.05; ** P < 0.01; ***P < 0.001.
